# Physical Implementation of Optical Material‐Based Neural Networks Processing Enabled by Long‐Persistent Luminescence

**DOI:** 10.1002/advs.202524334

**Published:** 2026-02-27

**Authors:** Sangwon Wi, Yunsang Lee

**Affiliations:** ^1^ Department of Physics and Integrative Institute of Basic Sciences Soongsil University Seoul Republic of Korea

**Keywords:** artificial synapse, long persistent luminescence, physical deep neural network, physical reservoir computing, synaptic behavior

## Abstract

Rapid advancements in artificial intelligence have magnified the inherent bottlenecks and energy inefficiencies of conventional von Neumann architecture. To address these limitations, processing information in a highly parallel, memory‐integrated manner mimicking the human brain, neuromorphic devices have emerged as a cornerstone of next‐generation computing. Among these, optical‐neuromorphic devices are particularly promising. By using light, they offer transformative advantages, such as high speed, massive bandwidth, and minimal signal interference. Accordingly, we propose long‐persistent luminescence (LPL) materials as novel substrates for optically operative artificial synapses. We utilize AGa_2_O_4_ (A = Mg, Ca, Sr, or Ba) luminescent oxides in which the intrinsic defect states enable excellent LPL properties without complex material engineering. Leveraging these properties, we demonstrate the physical implementation of optical material‐based neural processing, in which memory retention and nonlinear transformation are executed within the LPL material. As proof of concept, this physical neural network was applied to a real‐time Pong gameplay, demonstrating autonomous decision‐making through light‐driven signal processing. To further extend, we developed physical reservoir computing and physical neural network architectures. These architectures exploit the nonlinear temporal dynamics and luminescence mapping of LPL to perform handwritten digit recognition. Our findings establish LPL materials as a versatile platform for developing next‐generation, energy‐efficient optical‐neuromorphic systems.

## Graphical abstract

This study reports on the physical implementation of optical material‐based neural processing using long‐persistent luminescence as memory‐retention and nonlinear optical material. The system performs optical‐domain preprocessing with opto‐electronic interfaces for stimulus delivery and readout, enabling real‐time demonstrations including *Pong* gameplay and handwritten digit recognition.

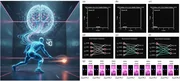

## 1 Introduction

Neuromorphic computing, which mimics the parallel processing capabilities of the human brain, has emerged as a compelling alternative [1, 2, 3, 4, 5, 6, 7]. In this paradigm, optically operated artificial synapses offer significant advantages in terms of speed, bandwidth, and energy efficiency [1, 2, 3, 4, 5, 6, 7]. However, realizing a practical and scalable photonic neuromorphic platform requires materials that can simultaneously perform optical excitation, memory retention, and dynamic signal modulation within a single system.

We propose and investigate a novel approach utilizing long‐persistent luminescence (LPL) materials as physical substrates for these optical synapses. The core of this approach is the unique photophysical properties of LPL. This phenomenon involves the trapping of excited electrons in intrinsic defect states and their subsequent thermal release, which results in a sustained afterglow. This intrinsic memory effect serves as a direct analog of biological synaptic plasticity [8, 9, 10, 11]. Crucially, the luminescence intensity and decay dynamics can be precisely modulated by controlling the parameters of the incident UV light pulses (e.g., intensity, duration, and frequency). This dynamic tunability allows LPL materials to emulate key synaptic functions in a purely optical domain [8, 9, 10, 11]. Despite these promising features, LPL materials have rarely been explored for neural computation. Previous optically driven synaptic devices, such as perovskite‐based phototransistors or memristive heterojunctions, often rely on complex hybrid architectures requiring electrical biasing or control circuitry, limiting scalability and real‐time performance. Here, we employ dopant‐free AGa_2_O_4_ (AGO, where A = Mg, Ca, Sr, or Ba) oxides, which exhibit excellent LPL under UV‐excitation [12, 13, 14, 15]. Exploiting this intrinsic persistence, we physically implement an optical material‐based neural network capable of real‐time neuromorphic computation. The system integrates optical excitation, memory retention, and signal readout within a single LPL‐based material platform. As a proof of concept, it performs real‐time decision‐making in a Pong gameplay task through light‐driven signal processing. Beyond this, we demonstrate the versatility of LPL‐based optoelectronic devices in realizing two key neuromorphic architectures: physical reservoir computing (PRC) and physical deep neural networks (PDNN) [1, 16, 17]. Leveraging the complex, non‐linear temporal dynamics and fading memory of LPL material, PRC efficiently transforms temporal pulse inputs into high‐dimensional spatiotemporal features [18, 19, 20]. Meanwhile, the precisely tunable luminescence states of LPL enable a physical activation function for hardware‐level inference in PDNNs, operating in a parallel manner. Furthermore, our study successfully demonstrates that potential drawbacks associated with residual luminescence are actively mitigated through the implementation of an IR‐assisted state‐reset protocol. This reset mechanism enables rapid de‐trapping of charge carriers and restoration of the baseline state between operations, effectively decoupling memory retention from response speed. Our demonstrations establish a scalable and fully photonic computing framework based on LPL materials, highlighting their potential as a versatile platform for real‐time, energy‐efficient, and optical material‐based neuromorphic intelligence.

## 2 Results

### 2.1 Photoluminescence and Long‐persistent Luminescence Properties of AGO

**FIGURE 1:**
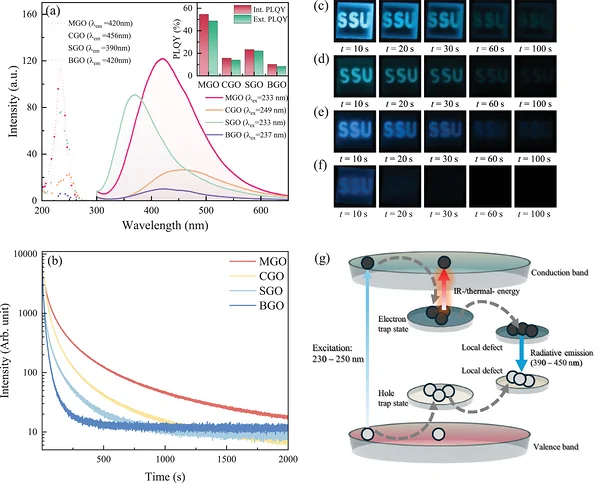
Photoluminescence (a) emission (solid lines) and excitation (dashed lines) spectra AGO. The inset plot in (a) represents photoluminescence quantum yields (PLQYs) of AGO samples. (b) Long persistent luminescence (LPL) after 5 min of UV‐light irradiation (λ = 254 nm) of AGO. Photographs of LPL from sheet shape (c) MGO, (d) CGO, (e) SGO, and (f) BGO composites captured at 10, 20, 30, 60, and 100 s after switching off the excitation, respectively. (g) Schematic energy level diagram of AGO.

Before discussing the synaptic functionalities, we first examined the photoluminescence (PL) characteristics of the AGO samples to establish their fundamental luminescent behavior. All the samples exhibited unique emission spectra under UV excitation. Figure 1a shows the PL spectra of the MGO, CGO, SGO, and BGO samples measured under UV excitation at λ_ex_ = 233, 249, 233, and 237 nm, respectively. Each sample exhibited broadband emission spanning a wide spectral range with different peak positions. The peak maxima of MGO, CGO, SGO, and BGO are centered at 420, 456, 390, and 420 nm, respectively [21, 22, 23, 24]. The corresponding CIE color coordinates derived from these spectra are shown in Figure S5. The internal and external photoluminescence quantum yields (PLQYs) of the AGO samples are presented in the inset of Figure 1a and Table S2.

The PL excitation spectra of AGO also revealed apparent excitation bands in the UV range (233, 249, 233, and 237 nm for MGO, CGO, SGO, and BGO, respectively), which were monitored at their corresponding emission maxima (Figure 1a). Note that the luminescence of AGO arises from electron–hole recombination in intrinsic defect states without any external dopants. Wide‐bandgap oxides naturally develop various point defects, such as oxygen vacancies (V_O_) or gallium–oxygen vacancy pairs ([V_Ga_−V_O_]) during high‐temperature synthesis. These defects introduce localized energy levels within the bandgap, which act as luminescence centers [14, 25, 26]. From the thermoluminescence (TL) properties, the trap depths were estimated to be 0.76 – 0.90 eV (Figure S7). A more detailed discussion of the LPL and TL of AGO is provided in Note S2.

Notably, the AGO samples exhibit robust LPL character, which serves as the essential property for realizing the optically operated synaptic functionalities explored in this study [12, 13, 14, 15]. After 5 min of UV light irradiation (λ = 254 nm), all the AGO samples exhibited intense and long‐lasting afterglow emission, as shown in Figure 1b. The decay curves featured notably prolonged tails, which are characteristic of strong LPL behavior. This persistent emission remained easily observable to the naked eye for several minutes in the dark (Figure 1c–f). This LPL behavior in AGO originates from the intrinsic trap states acting as electron and hole traps [27, 28, 29]. During UV excitation, electrons and holes are generated and subsequently captured by the defect trap states. After excitation ceases, the trapped charge carriers are thermally released and recombined radiatively at the luminescence centers (Figure 1g). The decay curves of LPL can be well described by an empirical power‐law model (nonlinear), reflecting scale‐invariant relaxation dynamics associated with distributed trap depths (Figure S6). The overall decay behavior is not strictly linear in the time domain and exhibits a controllable, weakly nonlinear temporal response governed by intrinsic trap‐mediated dynamics.

The broad distribution of intrinsic defect states provides a trap landscape that naturally generates nonlinearity and fading memory. Under above‐bandgap excitation, carriers progressively fill available traps. The shallow traps release carriers rapidly via thermal activation, while deeper traps act as longer‐lived reservoirs. After de‐trapping, carriers may recombine radiatively through emissive states or be re‐trapped by other defects with different depths and radiative efficiencies before eventually relaxing through emissive channels. This history‐dependent redistribution among multiple trapping and recombination pathways leads to a nonlinear, state‐dependent luminescence response that can be exploited for synaptic plasticity, reservoir‐type temporal processing, and activation‐like behavior without additional circuit elements [3, 4, 8, 30].

### 2.2 Fundamental Synaptic Behaviors of AGO: Pulse Timing‐/Duration‐based Signal Encoding

**FIGURE 2:**
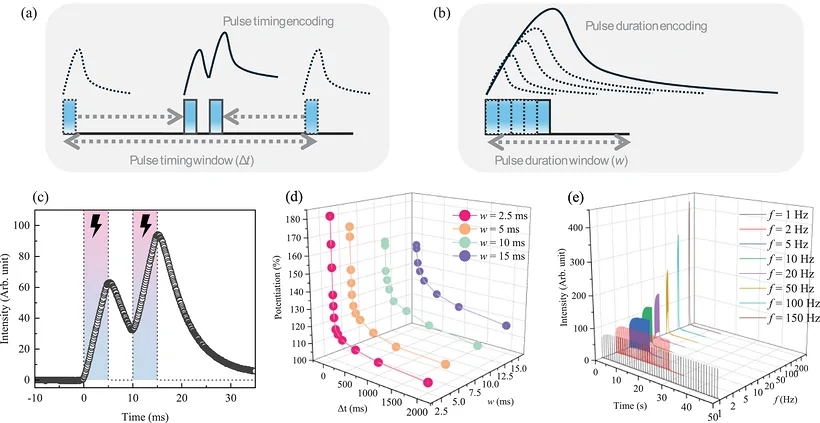
(a) A schematic for a signal encoding method using pulse timing tuning. (b) A schematic for a signal encoding method using pulse duration tuning. (c) Paired‐pulse facilitation (PPF) of MGO stimulated by UV‐pulses at *w* = 5 ms and Δ*t* = 5 ms. (d) PPF indexes of MGO with *w* varied 2.5 – 5 ms, and Δt varied 0.7 – 2000 ms. (e) Potentiation in multi‐pulse condition of MGO with 50 serial pulses (*n* = 50), *w* = 5 ms, and frequencies (*f*) varied 1 – 150 Hz.

The long, persistent character refers to the availability of intrinsic, light‐driven memory states of LPL whose effective temporal window is tunable and task‐dependent. By adjusting the excitation pulse parameters (timing, duration, and repetition), the system operates in a regime where the system provides short‐ to intermediate‐term optical memory. With the established fundamental luminescence mechanisms, we now demonstrate the synaptic functionality based on the underlying luminescence processes. The synaptic functionality, in essence, involves the conversion of introduced signals into analog responses that emulate biological synapses. To realize this behavior through LPL, we propose two fundamental approaches to achieve synaptic signal encoding. The first approach is the pulse‐timing encoding method, which utilizes the enhancement of the luminescence intensity by two sequential UV pulse excitations. As shown in Figure 2a, this concept is based on potentiation changes that depend on the time interval between the two UV pulses (Δ*t*). This approach, known as paired‐pulse facilitation (PPF), has generally been applied to several other synaptic systems [6, 7, 8, 9, 10, 11]. The second approach is a pulse‐duration‐based signal‐encoding method. As shown in Figure 2b, the maximum intensity of luminescence can be changed by adjusting the pulse duration (*w)*. In this manner, synaptic encoding can be programmed solely through optical control of excitation‐pulse timing and duration.

To validate these approaches, we examined the synaptic plasticity of our samples by varying both parameters: Δ*t* and *w*. We defined PPF as follows: PPF = (A_2_/A_1_) × 100%, where A_1_ and A_2_ represent the luminescence peak intensities of the two serial UV excitation pulses (Figure 2c) [6, 7, 8, 9, 10, 11]. This metric quantifies the pulse‐timing‐/duration‐dependent signal‐encoding capability of the system. As shown in Figure 2d, the luminescence of MGO exhibits apparent potentiation with a pair of UV excitation pulses at a given Δ*t* and *w*. To investigate the detailed dependence of Δ*t* and *w*, we evaluated the mapping of PPF with varying Δ*t* (Δ*t* = 1–2000 ms) and *w* (*w* = 2.5–10 ms) values. Under a fixed *w* with variable Δ*t*, a shorter Δ*t* yielded stronger potentiation. For example, at Δ*t* = 1 ms and *w* = 2.5 ms, the potentiation reached 181.57% but gradually decreased to 105.67% at Δ*t* = 2000 ms. Similar behaviors were also observed for other *w* conditions.

To examine device‐to‐device reproducibility, we prepared five additional MGO@PDMS samples (Note S3 and Figure S8) with similar geometry to the original sample. All supplementary samples showed comparable LPL emissions and nearly identical PPF characteristics under the same pulse condition, confirming robust stability and reproducibility.

We further examined the potentiation dependence of the multi‐pulse condition by changing the pulse frequency (*f*) of the introduced pulses (*f* = 1–150 Hz). Fifty excitation pulses with different *f* values were applied to each sample. Similar to the PPF case, as shown in Figure 2e, MGO showed an evident increase in potentiation as the frequency increased (i.e., a shorter time interval between the pulses). The maximum potentiation value increased from 130.3% to 663.5% as *f* was increased from 1 to 150 Hz. The synaptic behaviors of the other AGO samples were described in Note S4 and Figures S9–S11. Additionally, apart from this general synaptic behavior, the PPF values exhibit complicated dependence on Δt and w. We discussed more details of these dependencies in Note S4 and Figure S12.

We emphasize that the key functionality of our platform is not merely the linear superposition of time‐shifted luminescence transients, but a genuinely state‐dependent emission efficiency arising from nonlinear trap dynamics. This perspective is closely related to the recently introduced “Memlumor” concept, where the PLQY depends on excitation history and thus becomes formally analogous to an electrical memristor [31, 32]. In our AGO‐based system, excitation‐history‐dependent PLQY and trap‐mediated relaxation provide the physical origin of synaptic plasticity and nonlinear mapping, enabling task‐level neuromorphic demonstrations beyond a purely phenomenological description.

### 2.3 State‐reset Protocol Using IR‐irradiation on LPL‐based Synaptic Luminescence

We introduced an IR‐assisted state‐reset mechanism to ensure the reliable and reproducible operation of our LPL‐based synaptic system. We note that in parallel or repeated signal encoding, the residual LPL luminescence carried over from the previous operation can interfere with subsequent processing. Therefore, the implementation of an effective state‐reset mechanism is required for practical neuromorphic devices.

**FIGURE 3:**
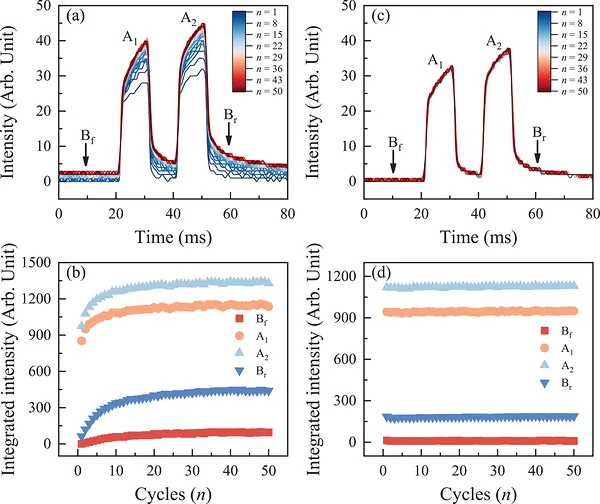
(a) Paired‐pulse facilitation (PPF) of MGO over 50 repeated cycles (*n* = 1 – 50) without IR‐reset (*w* = 5 ms and Δ*t* = 5 ms). (b) Forward baseline offset (B_f_), first peak (A_1_), second peak (A_2_), and residual background (B_r_) intensities from PPF of MGO without IR‐reset, as a function *n*. (c) PPF of MGO over 50 repeated cycles (*n* = 1 – 50) with IR‐reset (*w* = 5 ms and Δ*t* = 5 ms). (d) Changes of B_f_, A_1_, A_2_, and B_r_ intensities from PPF of MGO with IR‐reset, as a function *n*.

As shown in Figure 3a, we first examined the PPF behavior of MGO over 50 consecutive repetitions (*w* = 5 ms, Δ*t* = 5 ms, and *n* = 1–50) without any reset treatment. Both A_1_ and A_2_ increased with increasing *n*, and the residual background signal (B_r_) accumulated, acting as a forward baseline offset (B_f_) for subsequent operations. Consequently, as shown in Figure 3b, all the parameters drifted upward with increasing *n*. These results indicate that residual responses from prior operations lead to interference and cumulative bias during continuous operations.

To address this issue, we employed an IR‐assisted state‐reset process by applying infrared irradiation for 50 ms between consecutive cycles. In oxide ceramics, de‐trapped carriers can relax through multiple channels, including multi‐phonon‐assisted non‐radiative recombination at deep defect states, surface or interface‐related recombination centers, and defect clusters that act as efficient non‐emissive sinks [33, 34, 35]. In our experiment, we used a commercial 1050 nm IR‐LED. Introducing this IR illumination step effectively released the trapped carriers between cycles, thereby resetting the luminescent state. As shown in Figure 3c, 50 repeated PPF cycles with an IR state‐reset produced highly consistent and stable responses. Correspondingly, Figure 3d shows that all the parameters remained nearly constant with *n*, confirming the validity of our reset procedure. To further validate our state‐reset protocol using IR‐irradiation, we measured the variation in the PPF index as a function of randomized Δ*t* (Note S5 and Figure S13). The results demonstrate the robustness of our state‐reset protocol, which maintains consistent PPF indexes under repeated operations.

We note that our experimental results successfully demonstrate that IR irradiation reduces the luminescence to the baseline noise level. In the context of practical application, this realizes a functional reset. Experimentally, we observed that IR irradiation reduces the luminescence signal to the baseline noise level and suppresses history‐dependent interference in subsequent operations. This level of reset is sufficient for reliable neuromorphic operation and reproducible synaptic behavior, as demonstrated in repeated measurements.

### 2.4 Implementation of Physical Neural Networks for Real‐Time Pong Game Playing

**FIGURE 4:**
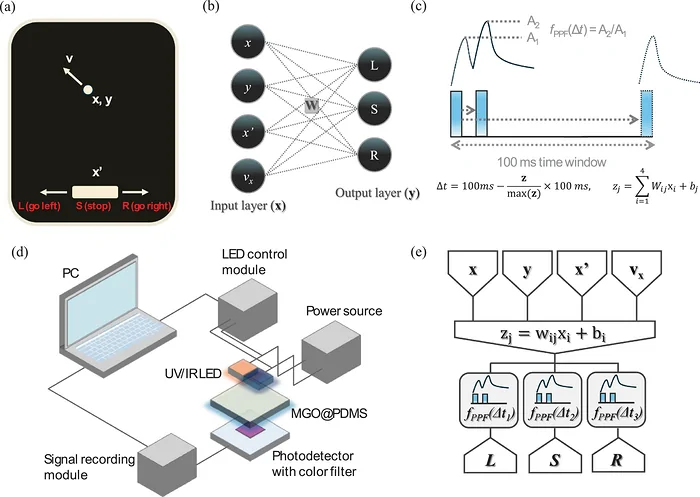
Schematics for (a) playing environment of Pong game, (b) Configuration of artificial neural network for Pong player, and (c) a concept for PPF based signal encoding (*f*_PPF_). (d) Operational configuration of Pong player PNN. (e) A schematic for parallel processing of *f*_PPF_.

Here, we demonstrate the implementation of a physical neural network (PNN) using an LPL‐based synaptic architecture. This architecture uses the change of LPL intensity in the material as a direct physical factor for a synaptic response after the activation function. We precisely program this by modulating the parameters (e.g., Δ*t* or *w*) of an incoming UV light pulse. The resulting peak emission is then optically read out as the synaptic output, forming the basis of our optical material‐based neural computation. To validate this practical implementation, we selected the classic video game Pong as a representative task owing to its simple structure, clear rules, and intuitive visualization [36]. The Pong game features a straightforward objective of scoring points by bouncing a ball, whereas the only controllable element is a paddle located at the bottom of the game display (Figure 4a). Although the game is simple, it requires real‐time decision‐making, making it an ideal task for validating the PNN functionality.

The PNN required for playing Pong can be implemented in a minimal configuration consisting of a simple 4 × 3 neural network without hidden layers (Figure 4b). The input layer comprises four nodes corresponding to the x‐ and y‐coordinates of the ball (x, y), the x‐coordinate of the paddle (x’), and the velocity along the *x*‐axis of the ball (v_x_). The output layer contains three nodes representing possible paddle movements: left (L), right (R), and stop (S). The weight and bias matrices for the neural network controlling the Pong player were pretrained by simulation on a PC using the PPF response curve obtained from the MGO sample. The process of our neural network can be expressed as [37]:

(1) $$z=\mathrm{Wx}+b,\;\;h_{j}=f_{PPF}\;\left(z_{j}\right)$$

(2) $$y=\mathrm{softmax}\left(h\right)$$

where **x** denotes the input vector, **W** and **b** are the weight matrix and bias vectors, respectively, **z** is the pre‐activation vector, **h** is the post‐activation vector obtained using *f*_PPF_, and *y* is the j‐th component of the output probability vector. As illustrated in Figure 4c, a time window is defined for the two excitation pulses. The ignition timings of the two pulses were modulated based on the input values such that higher input values resulted in the two pulses occurring closer in time (*f*_PPF_). For this, we directly converted components in **z** vectors to Δ*t* of PPF by normalizing the value to a range within 1–100 ms. Here, we note that in parallel or repeated signal encoding, residual luminescence from previous operations can interfere with subsequent processing. Implementing an effective state‐reset mechanism is highly required for practical neuromorphic devices.

The operational configuration of PNN is shown in Figures 4d and S14. An interface was constructed to manipulate the signals introduced into and received from the sample. The main integrated bay contained an integrated‐structured UV/IR LED‐MGO‐photodiode sensor module coupled with an LED control circuit, a signal recording board, and a power supply, all of which were interfaced with a MATLAB‐based control framework on a personal computer. Through this interface operation for playing Pong was executed, where each physical channel was independently processed, demonstrating the intrinsic parallelism of the PNN architecture (Figure 4e).

**FIGURE 5:**
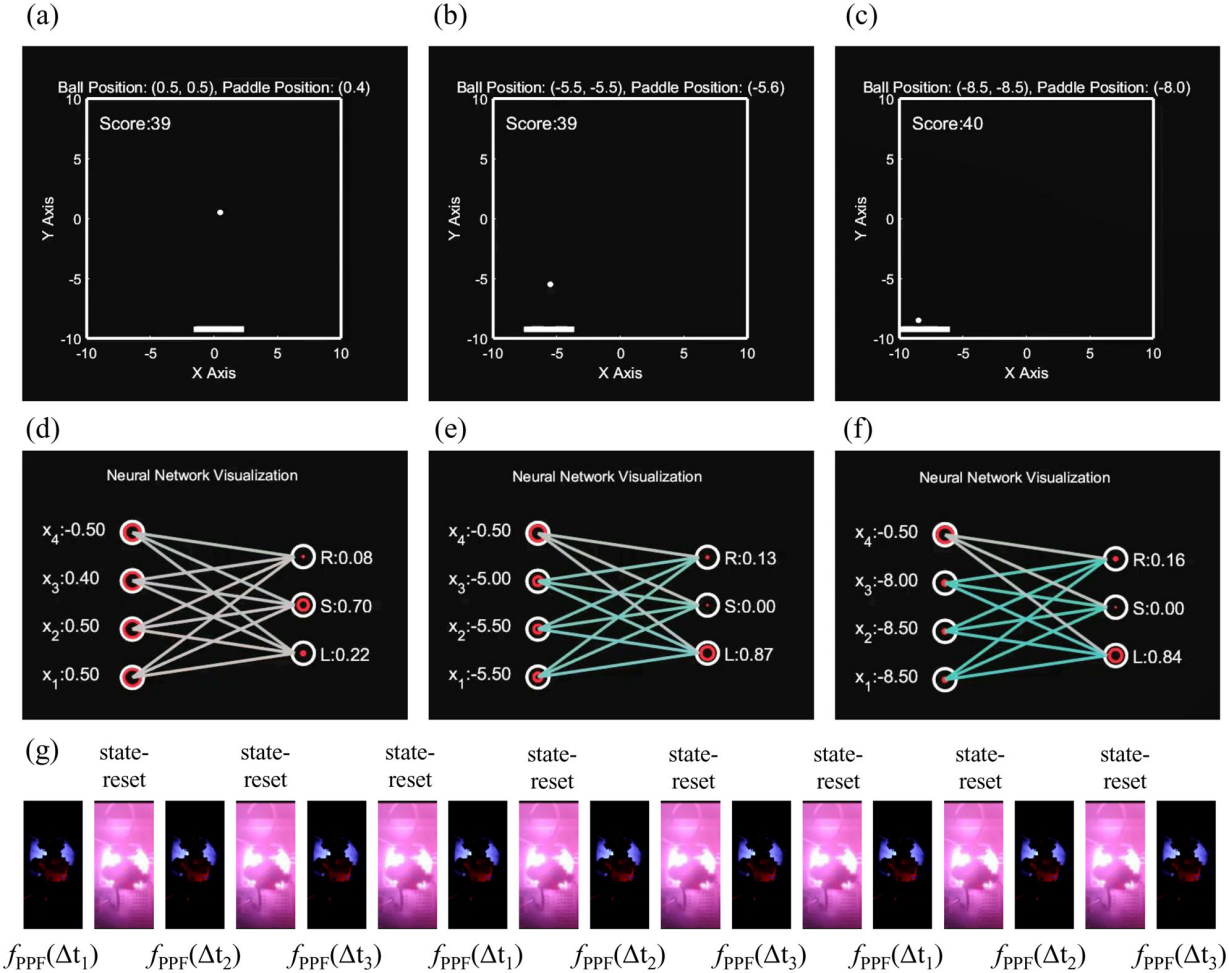
(a)–(c) Snapshots for the Pong game at selected moments where the game score from 39→40 (Time sequence: (a)→(b) →(c)). (d)–(f) The NN states at corresponding moments of (a), (b), and (c), respectively. (g) Photographs of the operating integrated bay.

A detailed operational sequence of the proposed PNN at a specific moment is shown in Figure 5. We captured the moments leading to the 40th scoring point during the real‐time decision‐making process of the PNN. Figure 5a–c depict snapshots of the game, and Figure 5d–f show the corresponding states of the neural networks. At the first moment, as the ball approaches from a distance (Figure 5a), the “S” (stop) output node exhibits the highest value, indicating the player's decision to keep the paddle stationary (Figure 5d). As the ball approaches (Figure 5b), the network reevaluates and decides to move to the left. Consequently, the value of the “S” node drops to zero, whereas the “L” (go left) node becomes dominant (Figure 5e). The network maintains this decision to move to the left (Figure 5f) until the paddle successfully intercepts the ball, resulting in a score (Figure 5c). Figure 5g shows the blinking emission photographs of the integrated bay during the sequence.

Video S1 shows real‐time Pong gameplay driven by our LPL‐based PNN. In the video, the top‐left panel displays the neural network architecture, the top‐right panel shows the Pong gameplay display, the bottom‐left panel shows the real‐time PPF signals obtained from the sample, and the bottom‐right panel shows a live recording of the PNN bay captured by a camera (8× accelerated for brevity). Consequently, the LPL‐based PNN successfully performed real‐time inference and achieved a final score of 100. Our results demonstrate the effective implementation of real‐time decision‐making using our LPL‐based PNN.

We note that the present prototype is not a fully optical neural network in the strict sense, because UV/IR LEDs and photodetectors are used as opto‐electronic interfaces for stimulus delivery and readout. Nevertheless, the core computational primitives, including history‐dependent memory, nonlinear transformation, and temporal integration, originate from optical‐domain dynamics within the LPL rather than from electronic current modulation. Therefore, our optical material‐based neural processing (or pre‐sensor/optical‐encoder) module coupled to digital post‐processing provides a clear route toward more integrated and parallel optical architectures [38, 39].

Recent studies have demonstrated that intrinsic luminescence dynamics (e.g., long‐persistent luminescence or up‐conversion luminescence) could support synaptic functions and reservoir‐type temporal processing [3, 4, 8, 30]. However, these prior reports primarily validated feasibility through materials characterization combined with software‐level modeling or offline processing, leaving a gap between device physics and system‐level operation. In contrast, the key contribution of the present work is the physical implementation and real‐time validation of an optical‐domain neural processing module based on LPL synaptic media. By directly using the measured material responses in an operational hardware setup, we demonstrate task‐level performance and clarify practical design constraints and engineering pathways toward scalable architectures.

### 2.5 Implementation of Physical Deep Neural Networks and Physical Reservoir Computing for Real‐Time Hand‐Written Digit Recognition

Subsequently, we expand our LPL‐based synaptic architecture to more advanced neuromorphic computing frameworks, specifically the PDNN and PRC. To verify this capability, we adopted the MNIST handwritten digit recognition task as a representative benchmark, given its wide use for assessing pattern recognition and classification in both conventional and physical neural networks [40]. We note that the scale‐invariant memory behavior of LPL is advantageous for reservoir computing. In RC, computational capability arises from a balance between fading memory and nonlinearity; a purely linear system would severely limit the richness of the reservoir state space. It allows the system to retain information over multiple timescales, unlike simple exponential decays, which lose information too quickly. This characteristic enhances the robustness of the reservoir against varying input frequencies.

**FIGURE 6:**
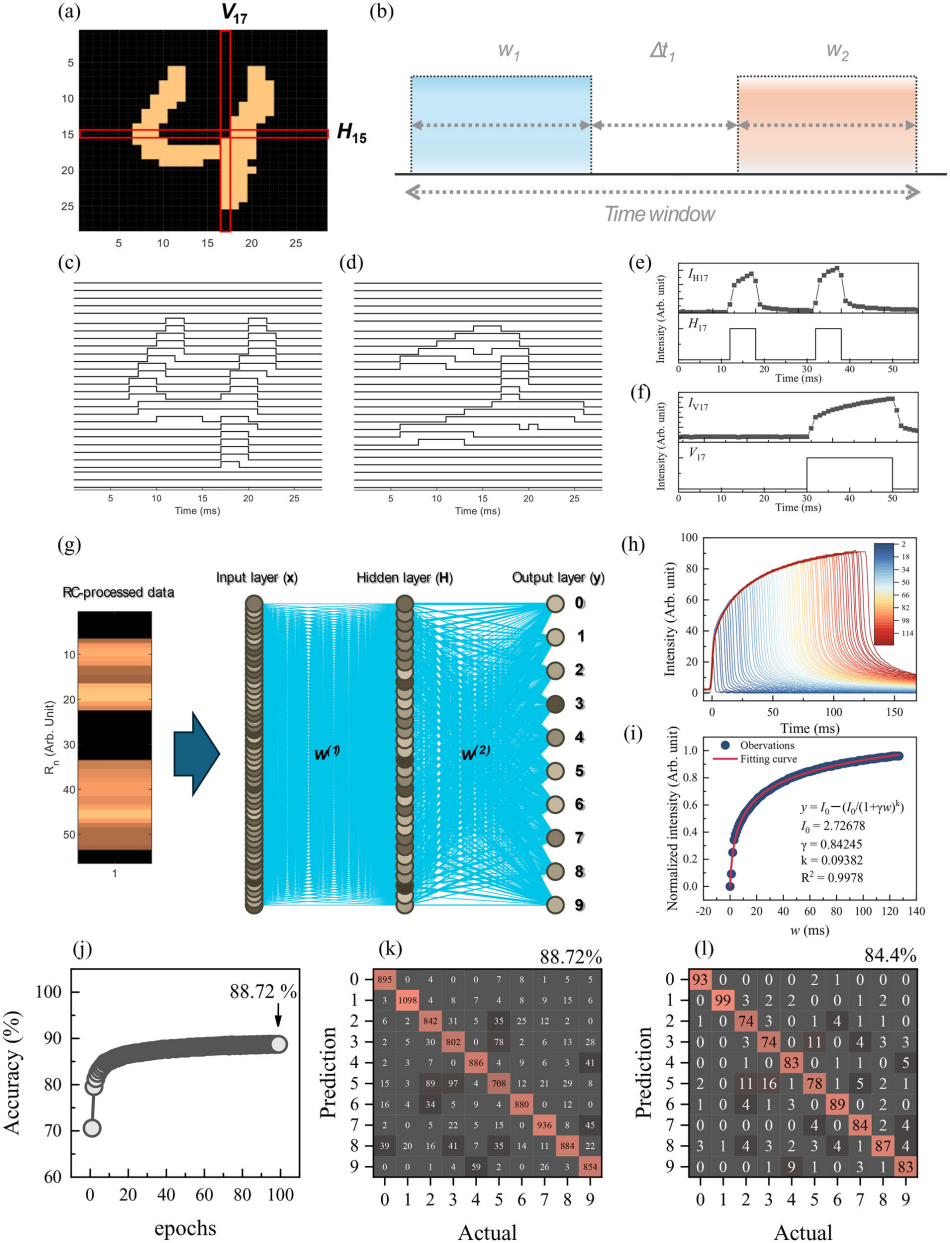
(a) An example of an input image (representing ‘4’). Red borders represent line extraction on the vertical or horizontal axis for the reservoir process. (b) A schematic of the introduced pulse sequence from *H*_15_ in (a). All pulse sequences from (c) *H*_n_ (n = 1 – 28) and (d) *V*_n_ (n = 1 – 28) of (a). Luminescence responses from the MGO by (e) *H*_15_ and (f) *V*_17_ pulse sequences. (g) Configuration of an artificial neural network for MNIST handwritten digit recognition with RC‐processing. (h) Pulse duration dependent luminescence response of MGO (*w* = 1 – 125 ms). (i) Fitted curve of luminescence response of MGO at *w* = 125 ms. (j) Accuracy of the neural network as a function of the training state in pre‐training. Heatmaps from the inferences by (k) simulated‐ and (l) real system‐PNN, respectively.

For the realization of PRC, we employed a simple approach that drives vertically and horizontally aligned feature extraction. As shown in Figure 6a, the handwritten image was composed of a 28 × 28 binary matrix. We extracted 28 vertical 1D data points (*V*_n_) and 28 horizontal 1D data points (*H*_n_). Subsequently, the processed image data were converted into on–off sequences within a predefined time window, as illustrated in Figure 6b [41, 42, 43]. Each pixel was mapped to a time slot of 2 ms, enabling the temporal encoding of spatial information. Using this method, we generated 56 distinct on–off sequences corresponding to the pixel columns, as shown in Figure 6c, d. These temporally encoded pulse sequences were then introduced into our LPL‐based synaptic architecture, resulting in unique LPL responses that reflect the spatiotemporal characteristics of the original input pattern. For example, Figure 6e, f show two on–off sequences (bottom panels) and the corresponding LPL responses (upper panels) of *H*_17_ and *V*_17_, respectively. By capturing the maximum intensity from each LPL response, the on–off sequences were transformed into a 1D vector (Figure 6g). We conducted this PRC process on the entire MNIST dataset, comprising 70 000 patterns (60 000 patterns for training and 10 000 patterns for validation), in preparation for the subsequent handwritten digit recognition task.

Building on this framework, we developed a PDNN for the real‐time recognition of handwritten digits. The PDNN represents a structural advancement over a simpler PNN. Although both architectures rely on physical signal encoding and analog computation through synaptic weighting, the PDNN incorporates one or more hidden layers to enable hierarchical feature extraction. This additional depth is critical for solving more complex classification tasks, where nonlinearity and abstraction are essential.

As shown in Figure 6g, we constructed the PDNN as a 58 × 32 × 10 neural network with input, hidden, and output layer structures. The PDNN was designed to be suitable for the PRC‐processed pattern data. The activation function in our PDNN was implemented using a pulse‐duration‐dependent signal encoding (*f*_PDE_) method, as described in Figure 2b. Therefore, the signal stream in the PDNN can be expressed as follows [37]:

(3) $$z^{\left(1\right)}=W^{\left(1\right)}\;x+b^{\left(1\right)},\;\;{h^{\left(1\right)}}_{j}=f_{PDE}\;\left(z_{j}^{\left(1\right)}\right)$$

(4) $$z^{\left(2\right)}=W^{\left(2\right)}\;h^{\left(1\right)}+b^{\left(2\right)},\;\;{h^{\left(2\right)}}_{k}=f_{PDE}\;\left(z_{k}^{\left(2\right)}\right)$$

(5) $$y_{k}=\mathrm{softmax}\left(h^{\left(2\right)}\right)$$

here, **x** denotes the PRC‐processed input vector; **W** ^(n)^ and **b** ^(n)^ are the weight matrix and bias vector of layer n, respectively; **z** ^(n)^ is the pre‐activation vector at layer n; **h** ^(n)^ is the post‐activation vector obtained using *f*_PDE_; and *y*_k_ is the k‐th component of the output probability vector. Superscripts (n) indicate the layer index (n = 1: input→hidden, n = 2: hidden→output), and all the vectors are column vectors.

Although the current network training and inference are implemented computationally, we deliberately use *f*_PDE_ derived from the measured material response to evaluate physical implementation feasibility. Using *f*_PDE_ rather than a generic ReLU/Sigmoid tests whether the intrinsic nonlinearity and saturation behavior of the LPL medium are sufficient to serve as a physical activation element in a future fully physical implementation. In this sense, *f*_PDE_ plays a conceptual role: it connects experimentally measured device physics to neural‐network functionality, demonstrating that the material response can support the required nonlinear mapping without relying on an algorithmic method. As shown in Figure 6h, we observed a highly repeatable *f*_PDE_ with *w* = 1–125 ms for the MGO sample. Interestingly, the luminescence buildup in *f*_PDE_ was accurately described by the complement of the function used to fit the LPL decay. This relationship can be expressed as:

(6) $$f_{PDE}\;\left(w\right)=I_{0}\;-I_{LPL}\;\left(w\right)=I_{0}\;-I_{0}/{\left(1+\gamma w\right)}^{k}$$

Here, *I*_0_, γ, and k are fitting constants, whereas *w* is the excitation pulse width. As shown in Figure 6i, this equation provides an excellent fit for the observed increase in dynamics. Notably, this power‐law saturating functional form is highly suitable as an activation function for artificial synapses. This nonlinear activation function enables deep neural networks to capture the nonlinear structure of real‐world problems, i.e., feature learning beyond simple linear mapping [44, 45]. We examined the feasibility of time‐integrated *f*_PDE_ by utilizing the full photon flux [32]. We experimentally evaluated integrated signals and found that the integrated response tended to be more linear with respect to pulse parameters (Figure S15), which is less suitable for the present demonstration, requiring a strongly nonlinear activation characteristic. During the pretraining of the PDNN, we used the fitting curve of *f*_PDE_ as an activation function. Backpropagation techniques were employed in the pretraining process. Our PDNN achieved an accuracy of 88.72% in simulation, after 100 epochs of training, as shown in Figure 6j, k. For quantitative performance estimation of our PDNN system, we conducted an additional hardware‐level validation by using 1000 validation datasets from MNIST, achieving an accuracy of 84.4% (Figure 6l).

Video S2 illustrates the complete operation of the inference process using our PRC and PDNN systems for handwritten digit recognition. In the video, instead of using a preloaded dataset, the digit “3” was manually written by the user as a real‐time input. The subsequent frames display six panels: the top‐left panel shows the input digit introduced into the system, the middle‐left panel shows the captured LPL responses, and the bottom‐left panel shows the live camera feed of the PDNN bay during operation. On the right side, the top‐right panel visualizes the temporal transformation of the input pattern by the PRC process; the middle‐right panel displays the activation of the hidden layer; and the bottom‐right panel displays the activation of the output layer. At the end of the video, the output node (bottom‐right panel) corresponding to the digit “3” exhibits the highest activation value, indicating that the system successfully recognized the input pattern.

In the present setup, a single LPL‐based synaptic device was used, and the 56‐dimensional feature vector was therefore acquired sequentially via time‐multiplexed excitation. As a result, the total inference time was limited by the pulse durations and the additional reset window. We employed a conservative time window of approximately 100 ms per excitation sequence, excluding the IR‐reset protocol, to ensure reliable signal discrimination and experimental reproducibility. Under this sequential configuration, the throughput was consequently low. Crucially, the proposed computational scheme is inherently compatible with spatial parallelism. In a scaled architecture consisting of an array of LPL‐based synaptic pixels, the 56 excitation sequences required for feature extraction could be delivered and read out in parallel, reducing the effective inference time per image to that of a single excitation–readout cycle.

Beyond the demonstrations presented here, it is important to consider the scalability, bandwidth, and efficiency of the proposed platform toward practical neuromorphic hardware. While the present study uses a single LPL element as a proof‐of‐concept building block, the material platform and operation principle are compatible with array‐based architectures for higher throughput and parallelism. Large‐scale optical integration and signal isolation strategies have been actively explored in recent premier studies [46, 47, 48]. Established strategies in optical computing and display technologies, such as pixel‐level optical confinement, micro‐apertures, absorptive or reflective isolation layers, and minimized emitter–detector spacing, can be directly applied to mitigate the crosstalk issue. In addition, spatially resolved excitation and detection schemes (e.g., patterned illumination or localized photodetector coupling) provide further degrees of freedom for isolating individual LPL nodes. While a quantitative characterization of spatial emission profiles and crosstalk suppression strategies is beyond the scope of the present proof‐of‐concept study, our results establish a modular and scalable building block, upon which array‐based architectures with appropriate optical isolation can be developed.

In the current prototype, we intentionally adopt a minimal operational configuration that primarily encodes information in the temporal amplitude dynamics of LPL. A wide range of LPL materials exists with emissions spanning from the ultraviolet to the near‐infrared, and wavelength‐selective excitation and readout schemes can, in principle, be integrated to realize spectral multiplexing and enhanced bandwidth. The current architecture, therefore, represents a minimal operational configuration, chosen to clearly isolate and validate the role of temporal luminescence dynamics in neuromorphic processing.

We note that the present proof‐of‐concept operates on a characteristic timescale on the order of ∼100 ms and uses relatively high LED power to secure sufficient signal‐to‐noise ratio in a non‐optimized bulk composite geometry. The current power budget is dominated by geometric and optical coupling losses in the prototype, and substantial reductions are expected through device miniaturization, improved optical confinement, and optimized emitter–detector coupling.

## 3 Discussion

In this study, we introduced LPL materials as novel physical substrates for optically operative artificial synapses and demonstrated the physical implementation of an LPL‐based synaptic architecture. We employed AGa_2_O_4_ (AGO, A = Mg, Ca, Sr, or Ba) luminescent oxides, which exhibit excellent LPL properties owing to their intrinsic defect states. The luminescence intensity of AGO can be precisely tuned by controlling the parameters of the UV excitation pulses, enabling a direct physical analog of the synaptic plasticity observed in biological synapses. We realized synaptic signal processing by encoding information into pulse‐timing‐ and pulse‐duration‐dependent LPL responses, enabling simultaneous optical synaptic operations and paving the way for physical deep neural networks (PNNs/PDNNs) and physical reservoir computing (PRC). Importantly, we demonstrate that possible limitations associated with residual luminescence are actively mitigated through an IR‐assisted state‐reset protocol, which accelerates de‐trapping and restores the baseline state between operations. As a result, the system maintains stable and responsive behavior during continuous operation by effectively decoupling memory retention from response speed. Building on this foundation, we successfully demonstrated hardware‐level inference by encoding synaptic weights into luminescence states. Notably, these LPL‐based PNN and PDNN architectures offer inherent parallelism, a key advantage that enables scalable neuromorphic systems. The performance of our LPL‐based systems was validated through representative tasks, including real‐time Pong gameplay and high‐accuracy MNIST handwritten digit recognition. In the Pong game, our optical material‐based PNN autonomously mastered the game and reached a perfect score of 100. Our combined PDNN and PRC architecture achieved an outstanding accuracy of more than 84.4% in the MNIST handwritten digit recognition task for physical operations. These findings establish that LPL‐based systems are not merely a proof‐of‐concept, but a versatile and powerful platform for developing the next generation of optical neuromorphic hardware.

## 4 Experimental Section

### 4.1 Materials and Synthesis

The AGa_2_O_4_ (AGO, where A = Mg, Ca, Sr, or Ba) samples were synthesized using a solid‐state reaction method. High‐purity MgO, SrCO_3_, CaCO_3_, BaCO_3_, and Ga_2_O_3_ were used as raw materials. The following ratio of raw materials was used: ACO_3_(MgO): Ga_2_O_3_ = 1:1. The mixed powders were milled and calcined at 500°C for 3 h and then sintered at 1300°C for 10 h. To embed the prepared powder in the polydimethylsiloxane (PDMS) matrix, PDMS (SYLGARD 184) and a curing agent were mixed and stirred in a beaker at a ratio of 3:7 to obtain a silica gel precursor. The AGO powder was mixed with the silica gel precursor in a weight ratio of 1:1. Subsequently, the mixture was slowly poured onto a square template until completely covered. The samples were allowed to stand in a vacuum chamber for 30 min to eliminate bubbles generated by stirring. Finally, the samples with the template were preheated to 50°C for 1 h for hardening.

### 4.2 Material Characterization

We acknowledge that the discussions of structural characterizations (XRD, Rietveld refinement, SEM, EDS) were provided in the Supporting Information. The X‐ray diffraction (XRD) patterns of the samples were measured using a Bruker AXS D2 system (Cu‐targeted X‐ray tube), and Rietveld refinement was performed to obtain detailed structural information regarding the samples (Note S1, Figure S1 and S2, and Table S1). SEM and EDS images were obtained using a Carl Zeiss Gemini SEM 300 system in the backscattering electron mode (Note S1, and Figures S3 and S4). The PL and PLE spectra were acquired using a spectrofluorometer (JASCO FP‐8500). The photoluminescence quantum yields (PLQY) were measured using an integrating sphere (JASCO ILF‐835) (Table S2). The UV‐pulse‐induced luminescence responses were recorded using a manually made detection system constructed with a function generator (YOKOGAWA FG300), a commercial UV LED with a 250 nm peak, and a high‐sensitivity light sensor (PASCO CI‐6604) covered with color filters to measure the emissions within a selective range. To demonstrate the PRC and PDNN, a commercial UV LED with a 250 nm peak was used as an excitation source, and a commercial IR LED with a 1050 nm peak was used for the state‐reset function. The UV‐ and IR‐LEDs were connected to IRLZ44N MOSFET transistors and an Arduino UNO module to introduce an on–off action to the LEDs. A TCS3200 photodiode light sensor and the Arduino UNO module were used to enable real‐time data acquisition using a PC. The integrated bay was structured in the order of UV‐ and IR‐LEDs/MGO/TCS3200 photodiode light sensors with a plastic guiding support structure (Figure S14). The PRC and PDNN operations were driven using a MATLAB‐based code framework.

## Conflicts of Interest

The authors declare no conflicts of interest.

## Supporting information




**Supporting File 1**: advs74628‐sup‐0001‐SuppMat.pdf

**Supporting File 2**: advs74628‐sup‐0002‐Video1.mp4

**Supporting File 3**: advs74628‐sup‐0003‐Video2.mp4

## Data Availability

The data that support the findings of this study are available from the corresponding author upon reasonable request.;
