# Supplementary material for: Physical Implementation of Optical Material‐Based Neural Networks Processing Enabled by Long‐Persistent Luminescence
Source: Adv Sci (Weinh). 2026 Feb 27;13(31):e24334. doi: 10.1002/advs.202524334 (PMC13588042; doi:10.1002/advs.202524334)
Supplement: Supplementary file 1 [file ADVS-13-e24334-s003.pdf]

Physical Implementation of Optical Material-Based Neural Networks Processing Enabled by Long-Persistent Luminescence

Sangwon Wi and Yunsang Lee

Department of Physics and Integrative institute of Basic Sciences, Soongsil University, Seoul 06978, Republic of Korea,

Supplementary Information

This PDF file includes:

- Supplementary Note S1, Figure S1–S5 and Table S1–S2
  : Sample characterizations of AGO

- Supplementary Note S2, and Figure S6–S7
  : Long-persistent luminescence and thermoluminescence of AGO

- Supplementary Note S3, and Figure S8
  : Stability and reproducibility of MGO@PDMS

- Supplementary Note S4, and Figure S9–S12
  : Comparison of synaptic behaviors among AGO samples

- Supplementary Note S5, and Figure S13
  : State-reset protocol using IR-irradiation on repeated PPF operations

- Supplementary Figure S14
  : Photographs of operational configuration

- Supplementary Figure S15
  : Time dependent luminescence intensity curve of MGO@PDMS

Note S1: Sample characterizations of AGO

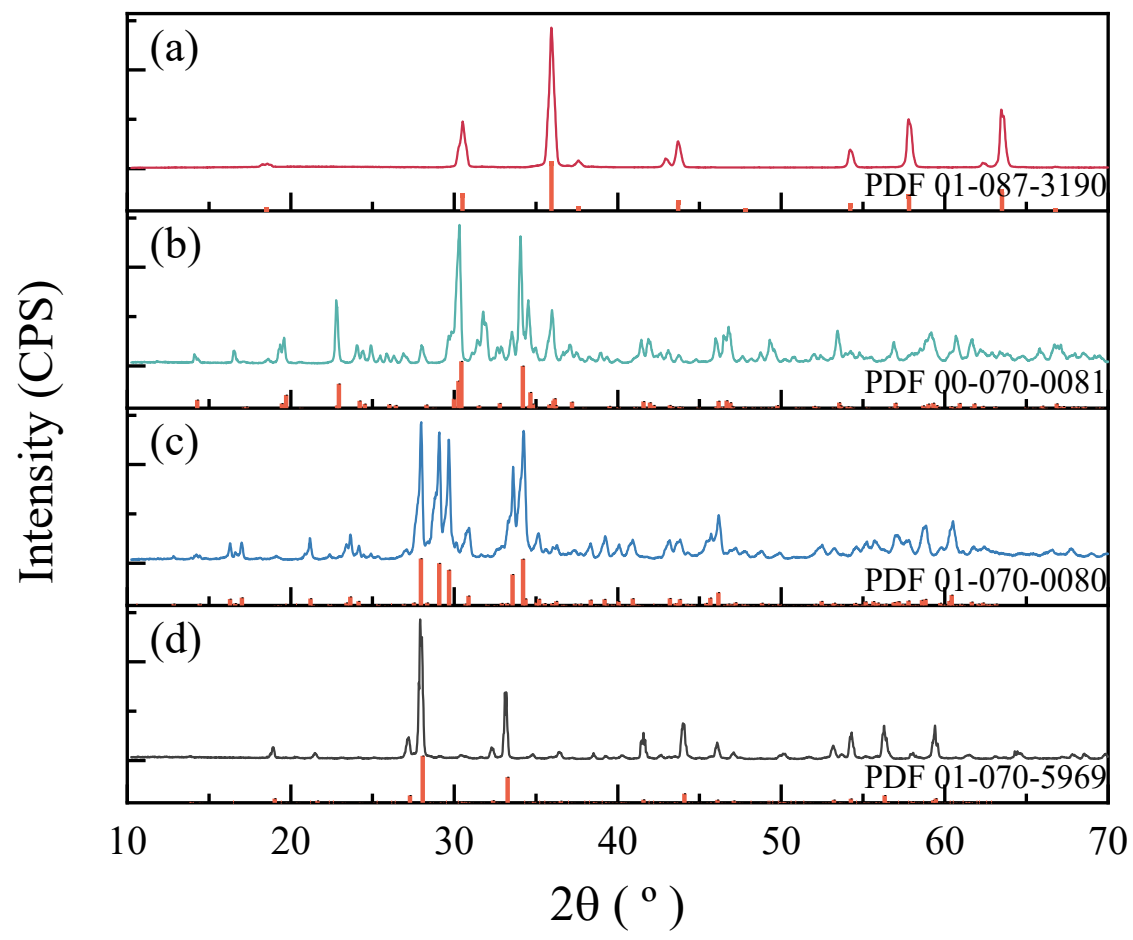

Figure S1. X-ray diffraction patterns of (a) MGO, (b) CGO, (c) SGO, and (d) BGO

X-ray diffraction (XRD) was performed to analyze the crystal structures of the synthesized AGO powders. Figures S1(a)–(d) show that the XRD patterns of all AGO samples closely match their corresponding reference patterns: $MgGa_2O_4$ (cubic (Fd-3m), PDF 01-087-3190), $CaGa_2O_4$ (orthorhombic ($Pna2_1$), PDF 00-070-0081), $SrGa_2O_4$ (monoclinic ($P2_1/c$), PDF 01-070-0080), and $BaGa_2O_4$ (hexagonal ($P6_3$), PDF 01-070-5969) [1-3]. These results confirm the successful synthesis of single-phase AGO compounds, each exhibiting a distinct crystal structure determined by the A-site cation.

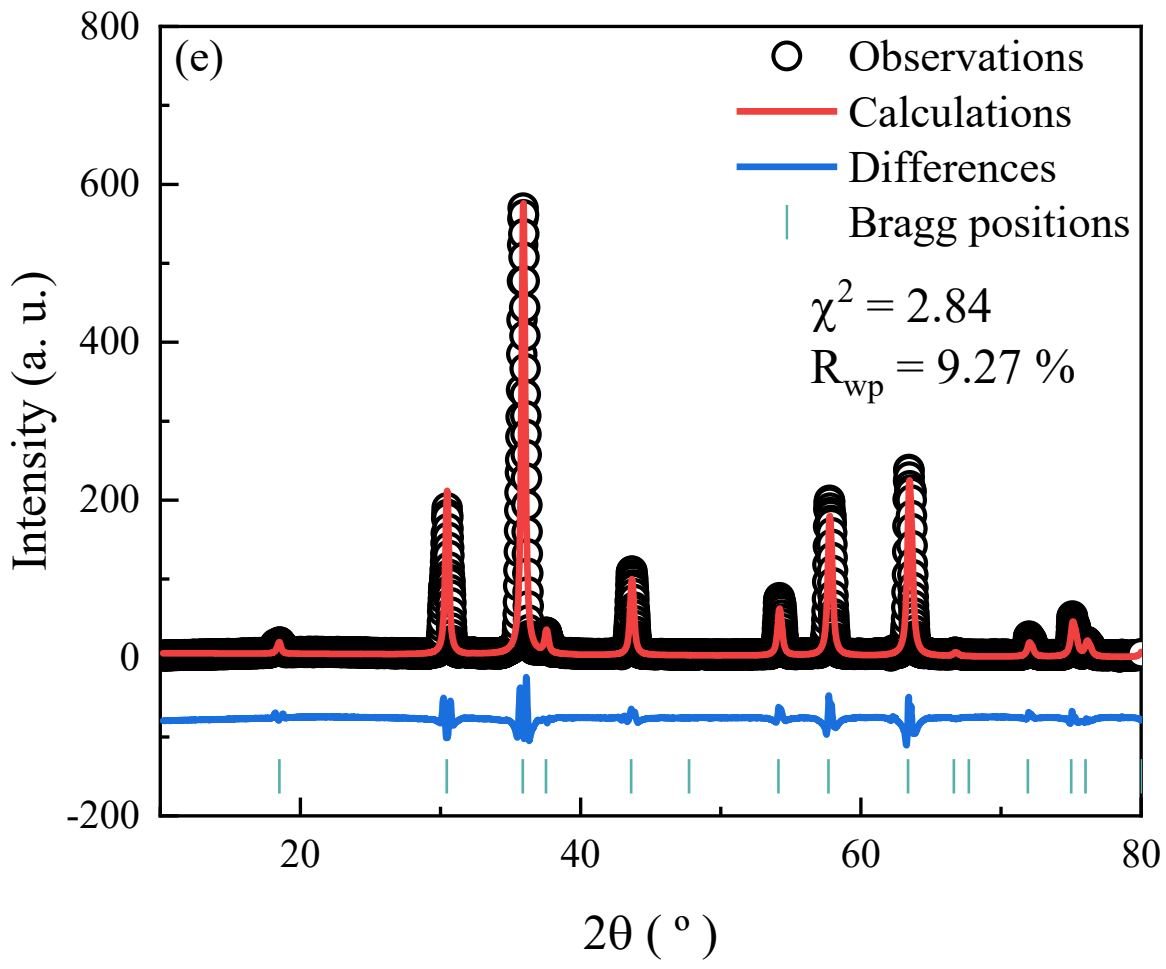

Figure S2. Rietveld refinement profile of MGO.

To obtain detailed structural information, we performed Rietveld refinement analysis on the XRD patterns (Figure S2) [4,5]. For the refinement, we utilized XRD patterns covering a 2θ range from 10° to 80° to ensure the inclusion of all major Bragg peaks. Appropriate space groups were assigned to each compound. Our refinement results showed weighted profile R-factors (R_{wp}) ranging from 12.55% to 20.16% and goodness of fit (χ^2) values below 4.89, indicating the acceptable reliability of our results [4,5]. Detailed refinement results, such as the refined lattice parameters, are listed in Table S1.

Table S1. Lattice parameters from Rietveld refinements of AGa_2O_4 (A = Mg, Ca, Sr, and Ba).

Sample	$MgGa_2O_4$	$CaGa_2O_4$	$SrGa_2O_4$	$BaGa_2O_4$
Structure	Cubic (Fd-3m)	Orthorhombic ($Pna2_1$)	Monoclinic ($P2_1/c$)	Hexagonal ($P6_3$)
a (Å)	8.295	10.363	8.382	10.744
b (Å)	8.295	7.751	8.996	10.744
c (Å)	8.295	9.129	10.670	8.650
α (°)	90.00	90.00	90.00	90.00
β (°)	90.00	90.00	93.86	90.00
γ (°)	90.00	90.00	90.00	120.00
Cell-volume ($Å^3$)	570.76	733.28	802.74	864.64
R_{wp} (%)	9.27	12.06	11.81	17.86
χ^2	2.84	3.00	3.15	3.99

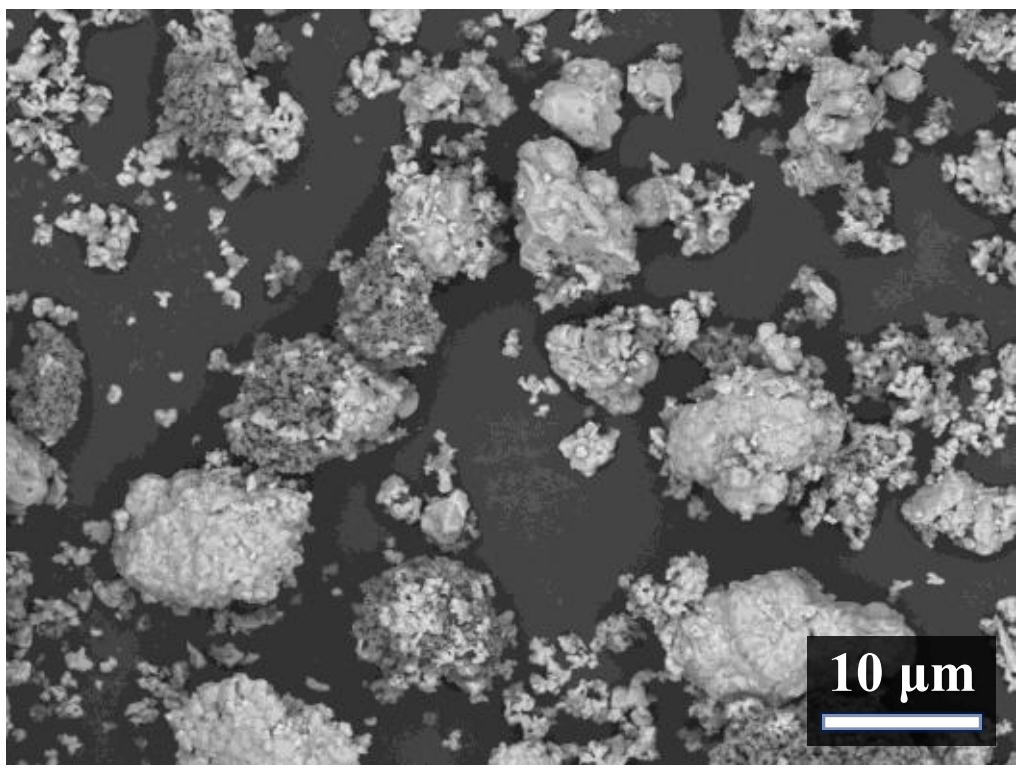

Figure S3. SEM image of MGO powder.

The microstructure and morphology of the AGO samples were investigated using scanning electron microscopy (SEM). Figure S3 shows the SEM image of the MGO sample. The micrometer-scale agglomerates, ranging from approximately 2 to 10 μm in size, are composed of smaller primary grains. These primary grains are submicron (less than 1 μm) and exhibit distinct, well-defined boundaries.

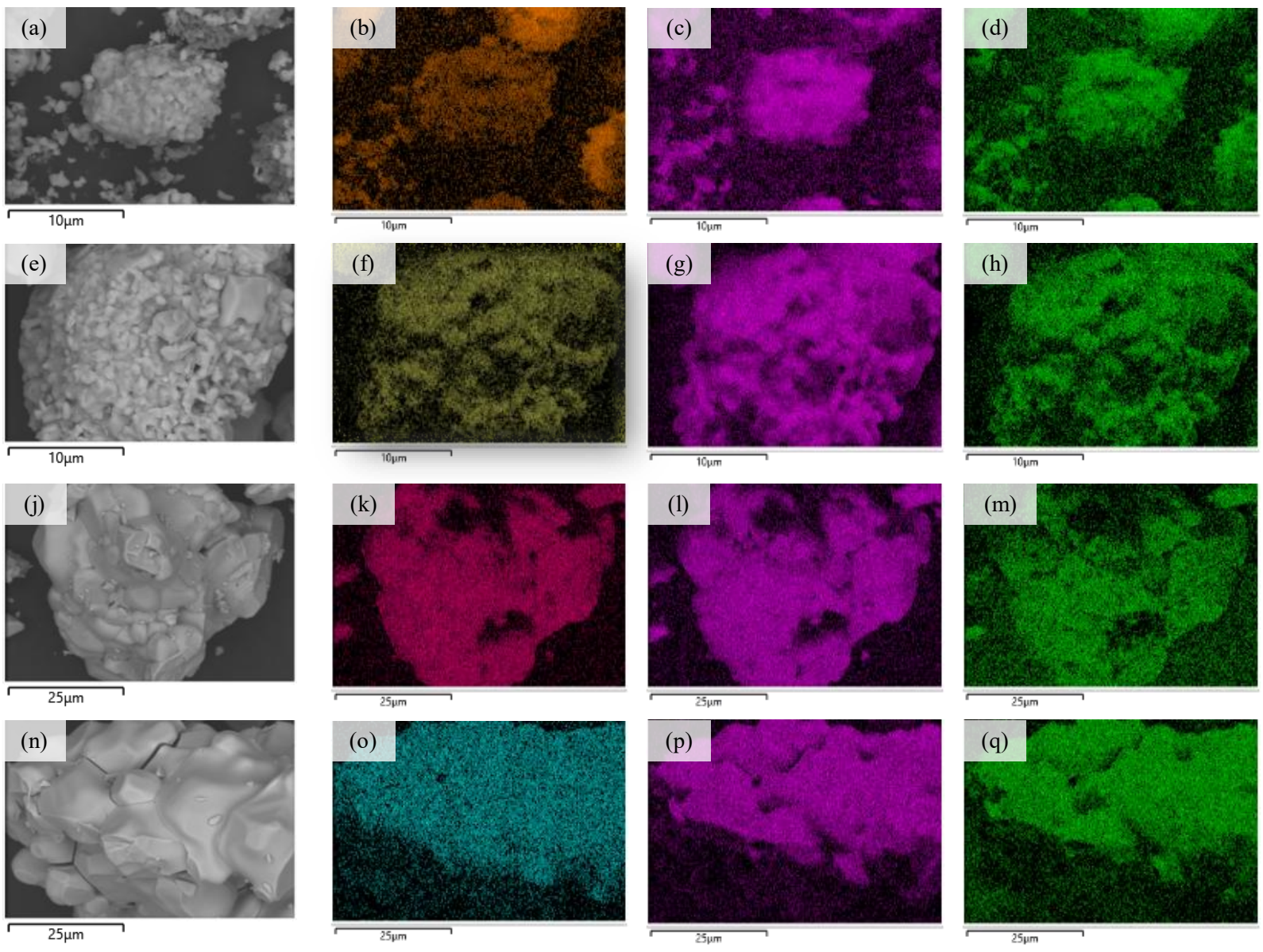

Figure S4. EDS mappings of AGO powders. (a) morphology of mapping area, (b) Mg, (c) Ga, and (d) O mappings of MGO. (e) morphology of mapping area, (f) Ca, (g) Ga, and (h) O mappings of CGO. (j) morphology of mapping area, (k) Sr, (l) Ga, and (m) O mappings of SGO. (n) morphology of mapping area, (o) Ba, (p) Ga, and (q) O mappings of BGO.

Energy-dispersive X-ray spectroscopy (EDS) mapping was performed to confirm elemental composition and distribution. The EDS mapping images in Figures S4(a)–S4(d) confirm that the constituent elements (Mg, Ga, and O) are uniformly distributed throughout the observed area of the MGO sample. The SEM images and EDS mappings of CGO, SGO, and BGO are shown in Figures S4(e)–S4(q). These results indicate the effective incorporation of all elements into the prepared samples, suggesting the excellent quality of the synthesized powders.

To enable reliable optical characterization and device integration, we fabricated device-compatible, sheet-like composites by embedding AGO powders into a polydimethylsiloxane (PDMS) matrix. PDMS was chosen for its excellent optical transparency, flexibility, chemical inertness, and particle dispersion properties [6,7]. All subsequent evaluations, including long-persistent luminescence (LPL), thermoluminescence (TL), and synaptic functionality, were performed using these sheet-shape composite films.

We measured the PL quantum yield (PLQY) of AGO to determine its luminescence efficiency. The internal and external PLQYs are summarized in Table S2. The internal PLQYs of MGO, CGO, SGO, and BGO were 54.60%, 15.72%, 23.30%, and 10.26%, respectively, and the corresponding external PLQYs were 48.71%, 14.11%, 22.14%, and 8.45%. The relatively high PLQYs of MGO, CGO, and SGO demonstrate that these materials possess sufficient optical efficiencies for potential optical applications. Consequently, the combination of high luminescence efficiency and dopant-free and facile synthesis highlights the exceptional potential of AGO as a practical and cost-effective luminescent material for various optical technologies.

Table S2. Photoluminescence quantum yield (PLQY) of AGa_2O_4 (A = Mg, Ca, Sr, and Ba).

sample	IQE (%)	EQE (%)
MGO	54.60	48.71
CGO	15.72	14.11
SGO	23.30	22.14
BGO	10.26	8.45

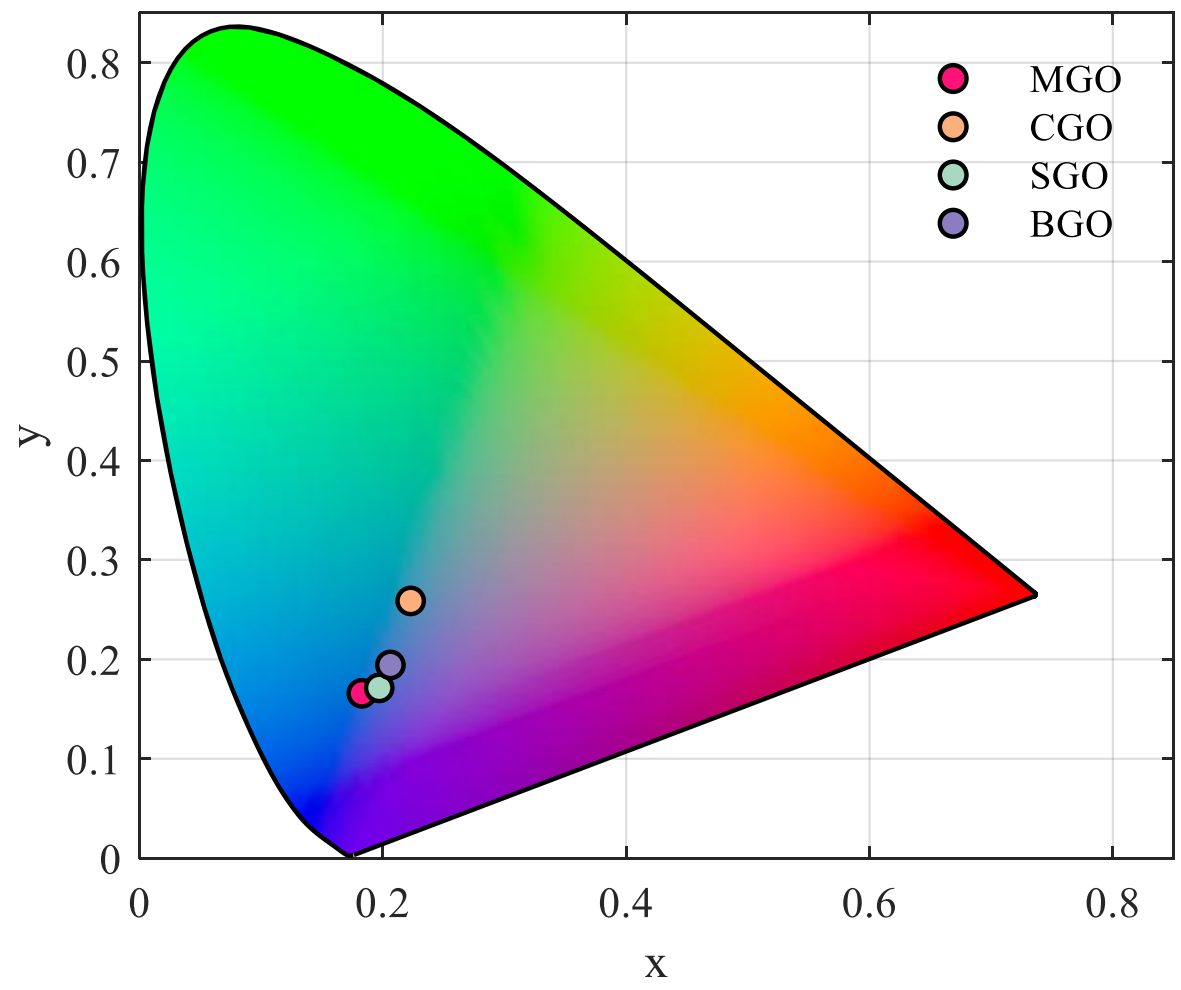

Figure S5. CIE coordinate map for PL spectra of AGO.

Note S2: Long-persistent luminescence and thermoluminescence of AGO

In LPL behavior of phosphor, a fraction of the trapped carriers can tunnel through the potential barrier to recombination centers, producing luminescence, which substantially retards the overall decay rate and prolongs emission. The contribution of the tunneling process between the trap and recombination states to the LPL decay can be quantitatively analyzed using the following empirical equation [8-12]:

$$I(t) = I_0/(1+\gamma t)^k \quad , \quad (1)$$

Here, $I(t)$ is the LPL intensity at time t, I_0 is the initial intensity, and γ and k are fitting parameters describing the trap-related recombination dynamics. The exponent k is directly related to retrapping dynamics, with values typically ranging from 0.5 to 2 [8-10]. A higher k value indicates steeper and shorter distance tunneling channels, leading to a faster decay rate. Conversely, a lower k value corresponds to a heavier-tailed tunneling distribution with substantial long-distance paths, resulting in slower and more persistent luminescence [8-10].

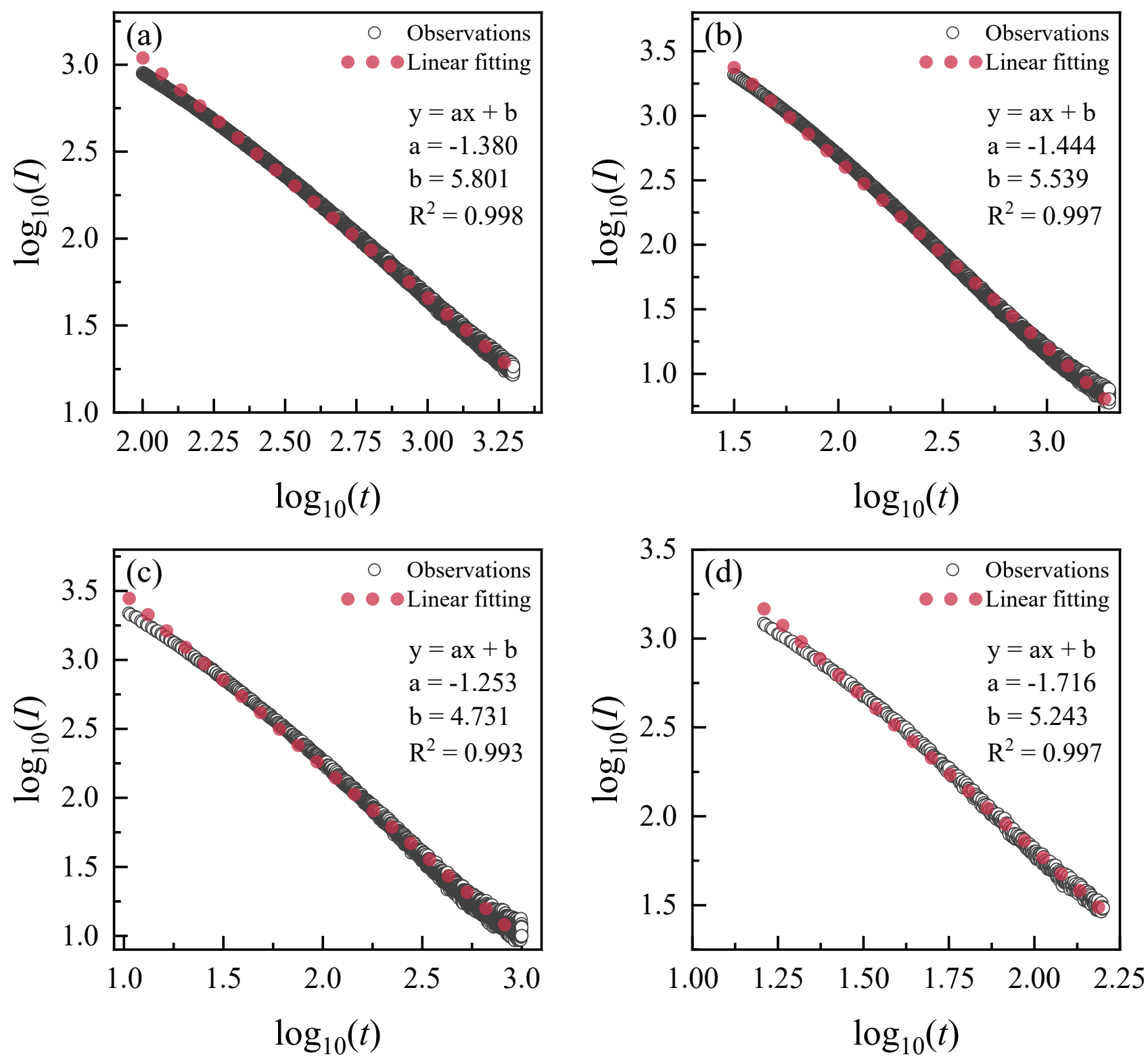

Figure S6. Long-persistent luminescence and fitting curves of (a) MGO, (b) CGO, (c)SGO, and (d) BGO within log-log scale.

As shown in Figure S6, we obtained the *k* values of our sample by linearly fitting the late-stage LPL curves plotted on a log-log scale. The fitted k values of MGO, CGO, and SGO were relatively small, i.e., 1.380, 1.444, and 1.253, respectively, whereas BGO exhibited a larger value of 1.716. The smaller k values of MGO, CGO, and SGO indicate a stronger retrapping process, which effectively prolongs recombination and leads to superior LPL compared with that of BGO.

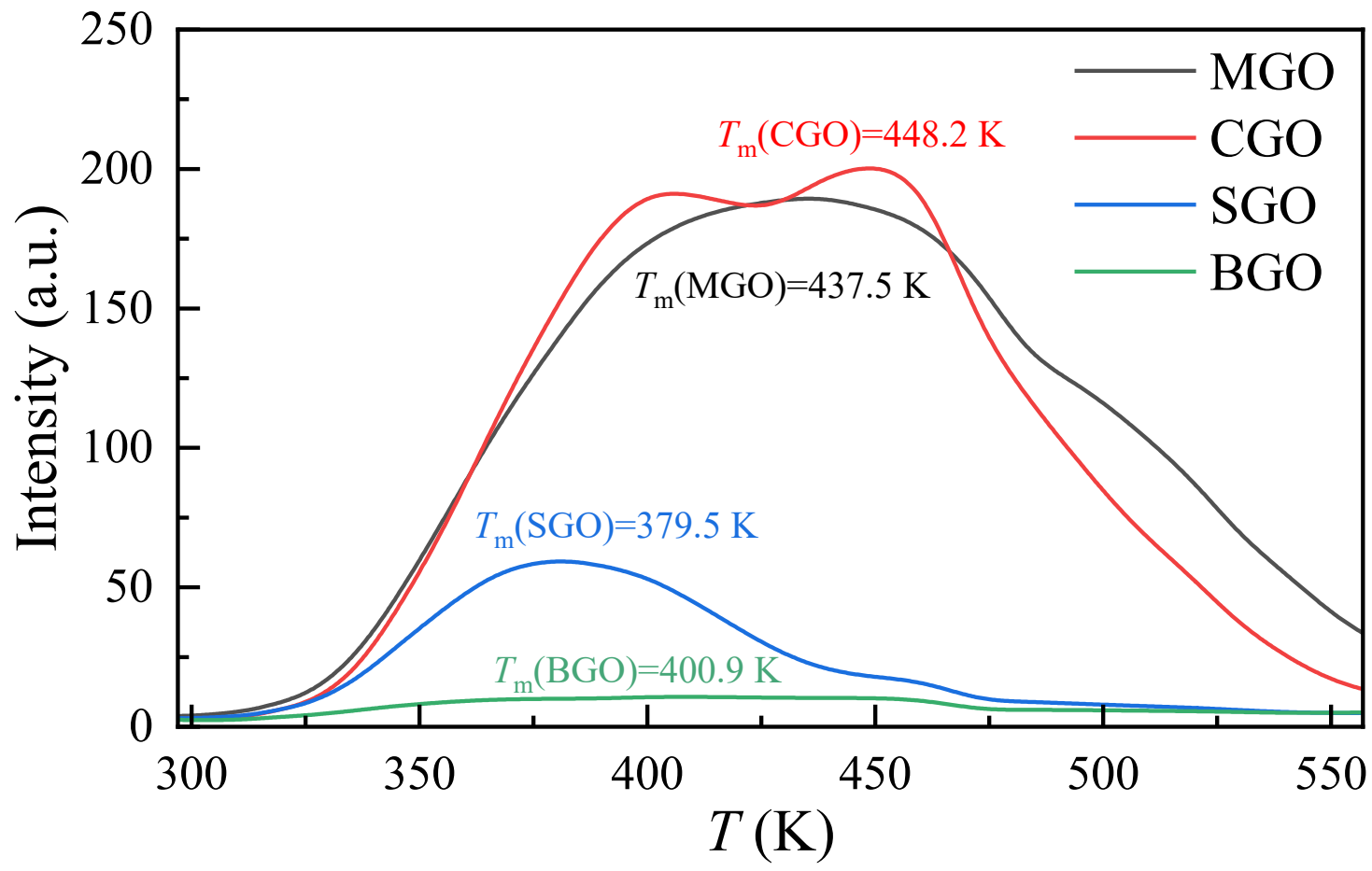

Figure S7. Thermoluminescence (TL) of AGO in range of 300 – 600 K with 0.5K/s of heating rate, after 5 min of UV irradiation (λ = 254 nm). T_m represents the temperature at maximum TL intensity of AGO samples.

To obtain detailed information on the defect states in our samples, we measured the TL of AGO after UV irradiation. As shown in Figure S7, the TL of the AGO sample exhibit prominent peaks in the range of 300–600 K. Each distinct peak in the TL spectra corresponds to a specific trapping center. Using a simple relation between the trap depth energy and TL, we calculated the specific trap depths (E_T) of AGO using the following equation [13-15]:

$$E_T = \frac{T_m}{500}, \quad (2)$$

where T_m is the temperature in Kelvin at the maximum intensity of the TL peak. The trap depth energies of MGO, CGO, SGO, and BGO were calculated to be 0.88, 0.90, 0.76, and 0.80 eV, respectively. These findings suggest that the AGO samples possess significant trap states that can store electrons under UV excitation.

Note S3: Stability and reproducibility of MGO@PDMS

Stability and reproducibility are indeed critical. LPL is an intrinsic bulk property governed primarily by defect-state energetics and trap distributions within the host lattice, rather than by the external morphology of individual micrometer sized powder particles. In this sense, variations in particle shape or micrometer-scale size have a limited impact on the fundamental LPL decay dynamics and memory behavior. This materials-intrinsic nature supports the robustness of the observed synaptic responses across different powder morphologies. To address this issue experimentally, we prepared five additional samples (Sample S1 – S5) with similar geometry to the original batch (Figure S8 (a)). We acknowledge that the MGO@PDMS sample used in synaptic operation is obtained by simple cutting from larger MGO@PDMS sheet. Therefore, supplementary MGO@PDMS samples are easily made by simple cutting from larger MGO@PDMS. The size of prepared MGO@PDMS samples are 5 × 5 mm^2, approximately, which is enough to cover photodiode sensor (width ≈ 1.25 mm) used in signal recording (Figure S8(a) – S8(b)). Furthermore, because of homogenous dispersion of MGO powder in PDMS matrix, the LPL responses from the samples are similar, as shown in Figure S8(c) – S8(d). The PPF responses from these samples showed high consistency (Figure S8(e)), confirming the reproducibility of our device performance.

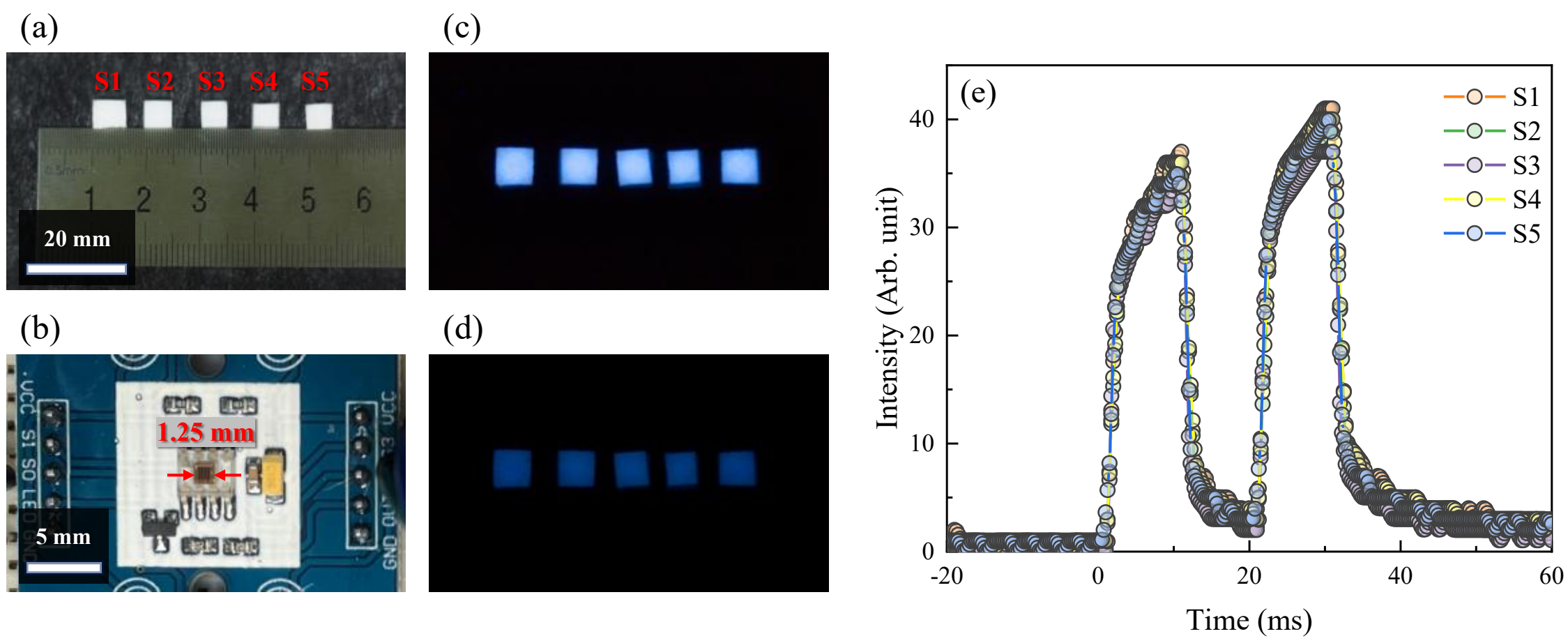

Figure S8. Photographs of (a) supplementary MGO@PDMS samples (Sample S1 – S5), (b) photodiode sensor used for signal recording, (c) luminescence of MGO@PDMS samples under UV, and (d) LPL of MGO@PDMS samples after UV-off, respectively. (e) PPFs (Δt = 5 ms, w = 5 ms) of sample S1 – S5.

Note S4: Comparison of synaptic behaviors of AGO

We observed that, similar to MGO, the CGO, SGO, and BGO samples also exhibited clear dependences on Δt, w, and f, as shown in Figures S9–S11. The comparison of key metrics, such as the PPF index at $\Delta t = 1$ ms, the PPF index at $\Delta t = 2{,}000$ ms, and maximum potentiation in multipulse excitation, among the AGO series is shown in Figures S12. Although all the AGO samples exhibited similar qualitative trends, MGO showed the most pronounced contrast and the highest optical efficiency. Considering its large signal modulation range and superior PLQY, MGO was selected as a suitable representative sample to demonstrate the PRC and PDNN.

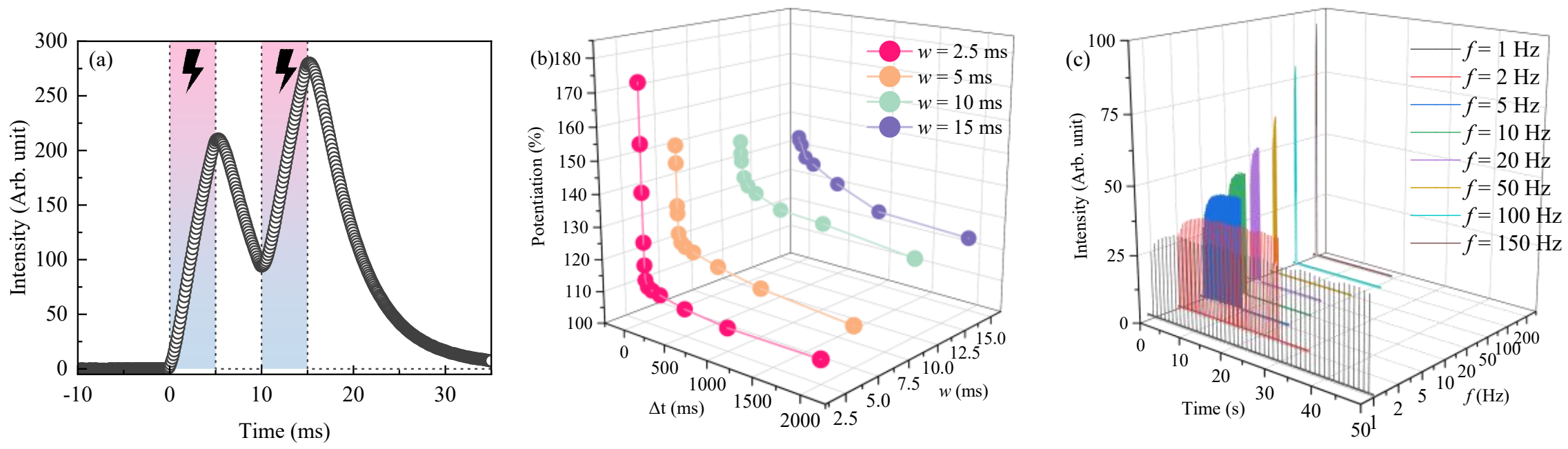

Figure S9. (a) Paired-pulse facilitation (PPF) of CGO stimulated by UV-pulses at $w = 5$ ms and $\Delta t = 5$ ms. (b) PPF indexes of CGO with w varied from 2.5 to 5 ms and Δt varied from 0.7 to 2,000 ms. (c) Potentiation in multi-pulse condition of CGO with 50 serial pulses (n = 50), $w = 5$ ms, and frequencies (f) varied from 1 to 150 Hz.

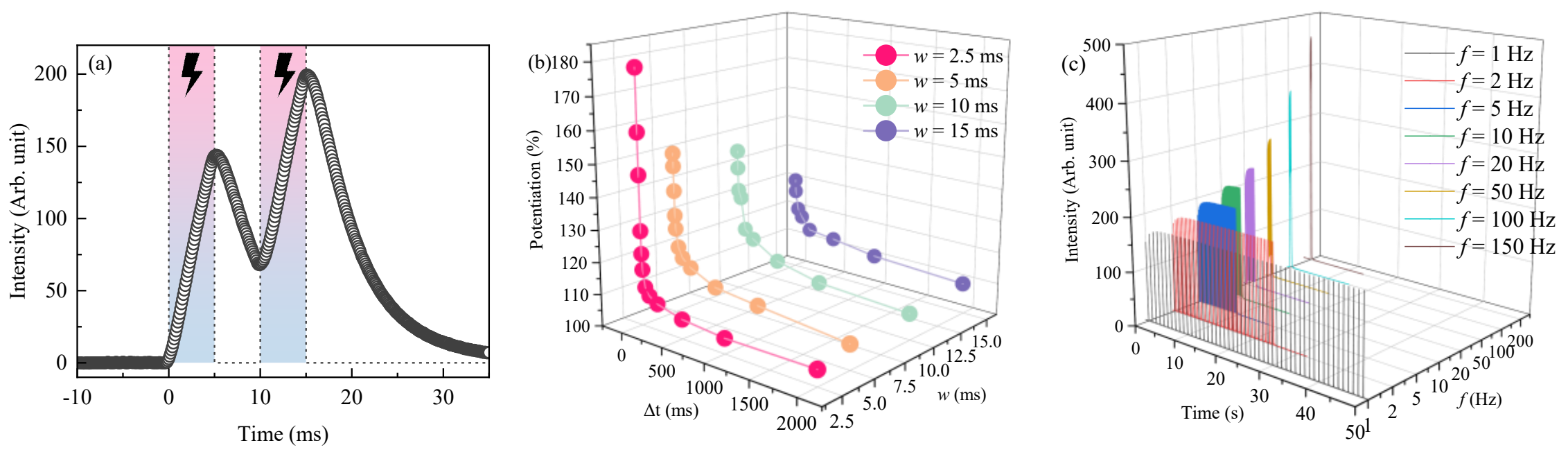

Figure S10. (a) Paired-pulse facilitation (PPF) of SGO stimulated by UV-pulses at $w = 5$ ms and $\Delta t = 5$ ms. (b) PPF indexes of SGO with w varied from 2.5 to 5 ms and Δt varied from

0.7 to 2,000 ms. (c) Potentiation in multi-pulse condition of SGO with 50 serial pulses (n = 50), w = 5 ms, and frequencies (f) varied from 1 to 150 Hz.

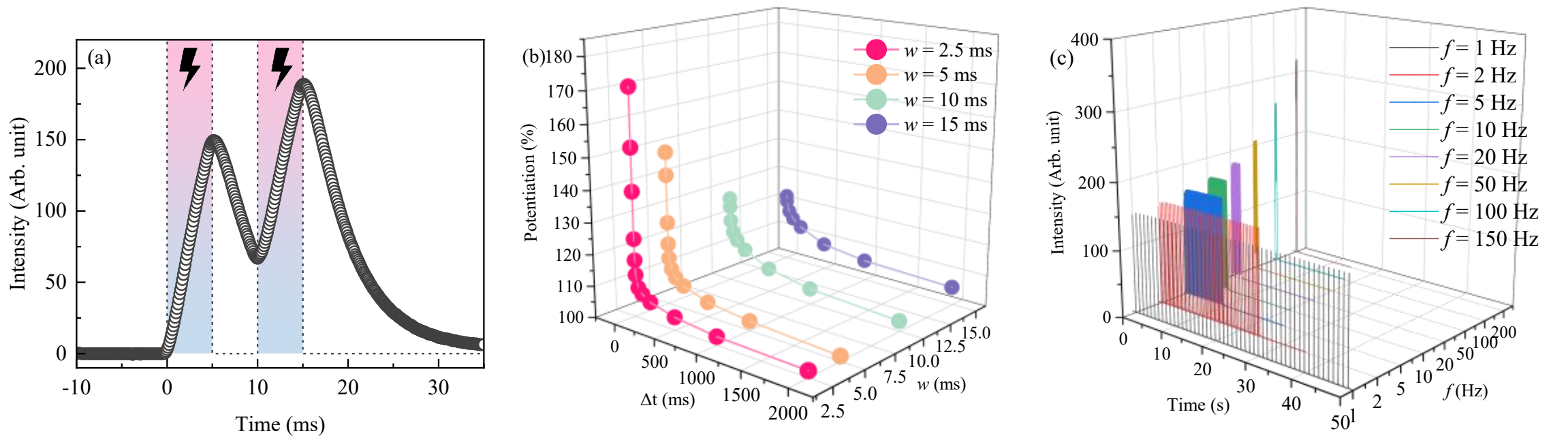

Figure S11. (a) Paired-pulse facilitation (PPF) of BGO stimulated by UV-pulses at w = 5 ms and Δt = 5 ms. (b) PPF indexes of BGO with w varied from 2.5 to 5 ms and Δt varied from 0.7 to 2,000 ms. (c) Potentiation in multi-pulse condition of BGO with 50 serial pulses (n = 50), w = 5 ms, and frequencies (f) varied from 1 to 150 Hz.

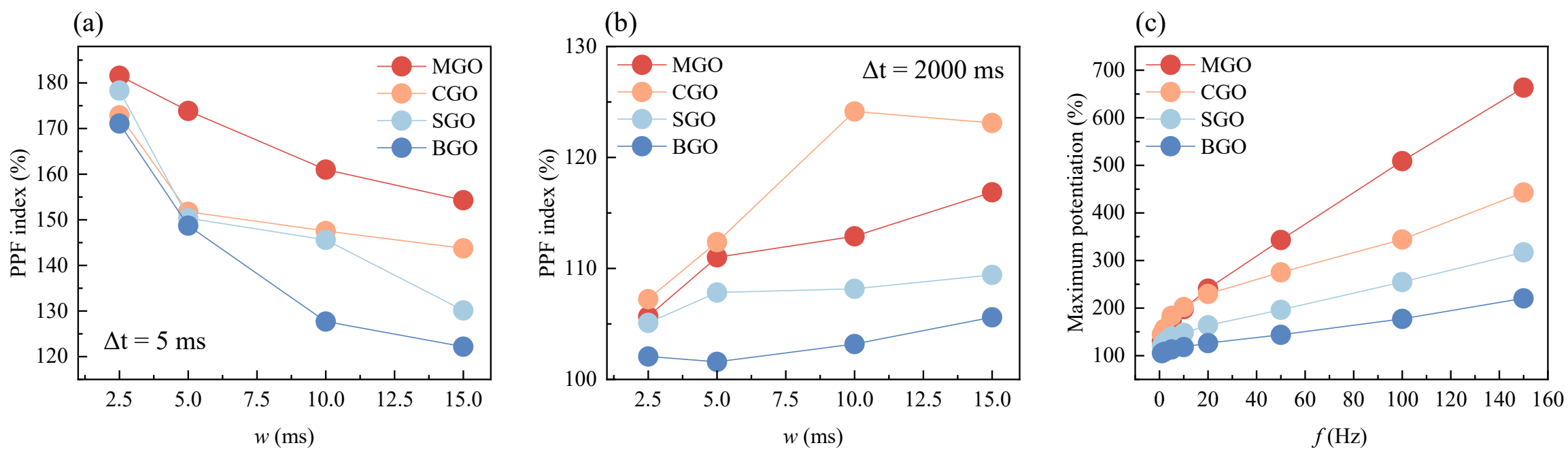

Figure S12. (a) Maximum PPF indexes of AGO as a function of w. (b) PPF indexes at Δt = 2,000 ms of AGO as a function of w. (c) maximum potentiation of AGO under a multi-pulse condition (n = 50) as a function of f.

Interestingly, at fixed Δt = 1 ms (Figure S12(a)), the PPF values decreased with increasing w (For MGO, 181.6%, 173.9%, 161.0%, and 154.8% at w = 2.5, 5.0, 10, and 15 ms, respectively), whereas the opposite trend appeared at Δt = 2,000 ms (Figure S12(b)), where the PPF increased with longer w (For MGO, 105.7%, 111.0%, 112.9%, and 116.9% at w = 2.5, 5.0, 10, and 15 ms, respectively). We attributed these contrasting trends to two competing mechanisms that are widely observed in phosphors. The first is the emission saturation. Under constant excitation, luminescence follows a saturating buildup. Consequently, as w increases,

the marginal gain in the emissions from the first pulse diminishes. At a very short Δ*t*, this saturation limits the relative increase in the second peak, causing the PPF to decrease as *w* increases. The second mechanism is the interpulse carryover. A longer *w* generates more intense persistent luminescence that survives the pulse interval. When Δ*t* is long, this residual signal increases the baseline of the second pulse. This effectively increases the amplitude of the second peak, thereby increasing the PPF as *w* increases.

Note S5: State-reset protocol using IR-irradiation on repeated PPF operations

To further validate our state-reset protocol using IR-irradiation, we measured the variation in the PPF index as a function of randomized Δ*t* (Figures S13(a)–(b)). A strictly ordered Δ*t* operation can impose a straight-ordered background bias, potentially producing correlated interference. In contrast, randomizing Δ*t* turns the carryover into stochastic noise. As shown in Figure S13(a), the PPF curve without the state-reset step exhibits strong noise under randomized Δ*t*, whereas applying the IR reset markedly suppresses this fluctuation (Figure S13(b)). Therefore, IR irradiation provides a robust and consistent state-reset mechanism that effectively eliminates residual luminescence and enables the reliable parallel processing of synaptic signal encoding in LPL-based synaptic systems.

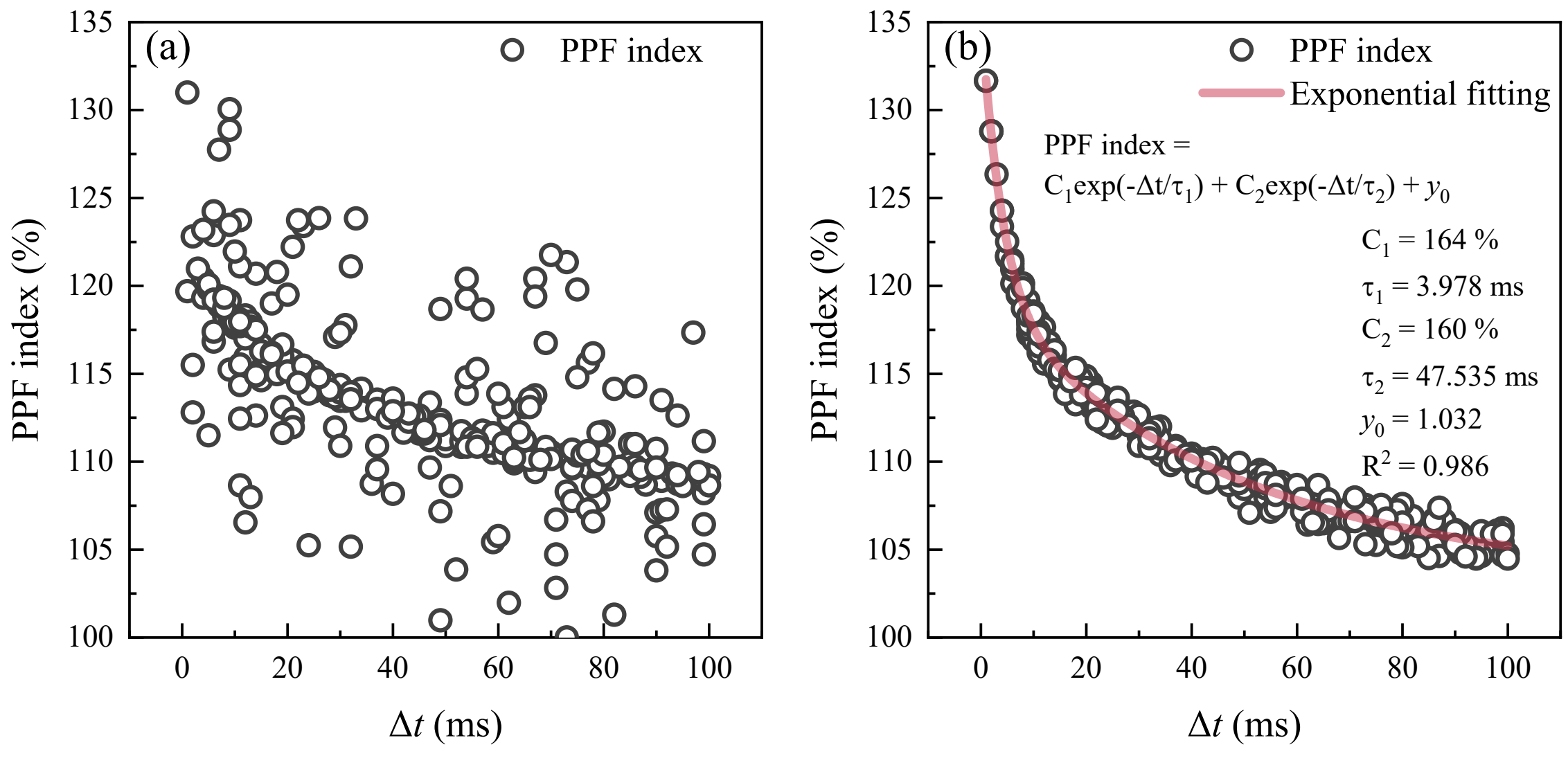

Figure S13. (a) PPF indexes from MGO by 1,000 of random Δ*t* without IR-reset (*w* = 5 ms, and Δ*t* = 1 – 100 ms range, random sequence). (b) PPF index curve from MGO by 1,000 of random Δ*t* with IR-reset (*w* = 5 ms, and Δ*t* = 1 – 100 ms range, random sequence)

Accordingly, the PPF index as a function of Δt is well described by a bi-exponential decay with fast and slow components [16-18]:

$$PPF\text{ index} = C_1 \exp\left(\frac{-\Delta t}{\tau_1}\right) + C_2 \exp\left(\frac{-\Delta t}{\tau_2}\right) + y_0 \quad , \qquad (3)$$

where t is the interpulse interval, C_1 and C_2 are the facilitation amplitudes, τ_1 and τ_2 are the corresponding relaxation time constants, and y_0 is the constant offset. The facilitation amplitudes were obtained as C_1 = 164%, and C_2 = 160%, whereas the relaxation time constants were obtained as τ_1 = 3.978 ms and τ_2 = 47.535 ms. Consequently, the memory effect of the pre-pulse on consecutive pulses can notably intensify when the pulse interval is short; this trend is in accordance with observations in biological synapses.

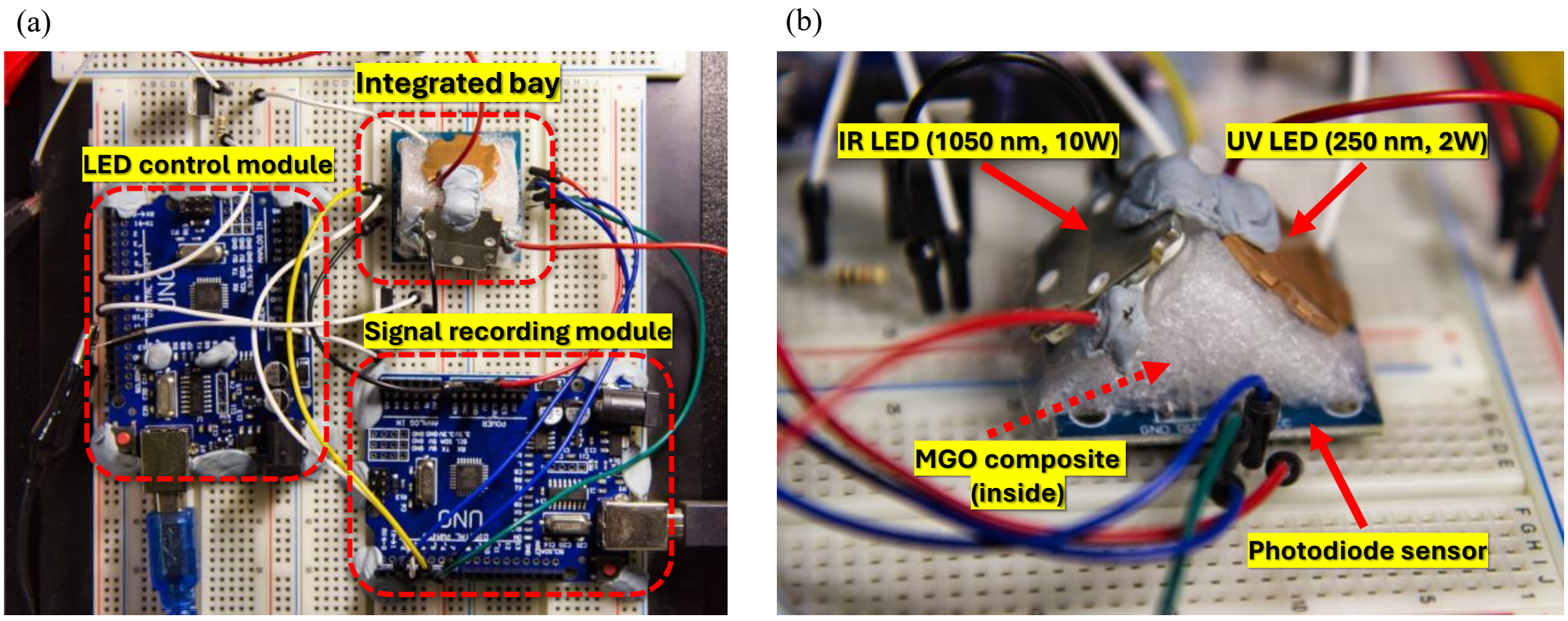

Figure S14. Photographs of experimental setup for LPL based PNN. (a) Configuration of integrated bay, LED control module, and signal recording module. (b) Configuration of integrated PNN bay which is combined by IR-LED, UV-LED, photodiode sensor, and sheet-shape MGO composite (sheet-shape MGO composite is packaged into the plastic cover inside).

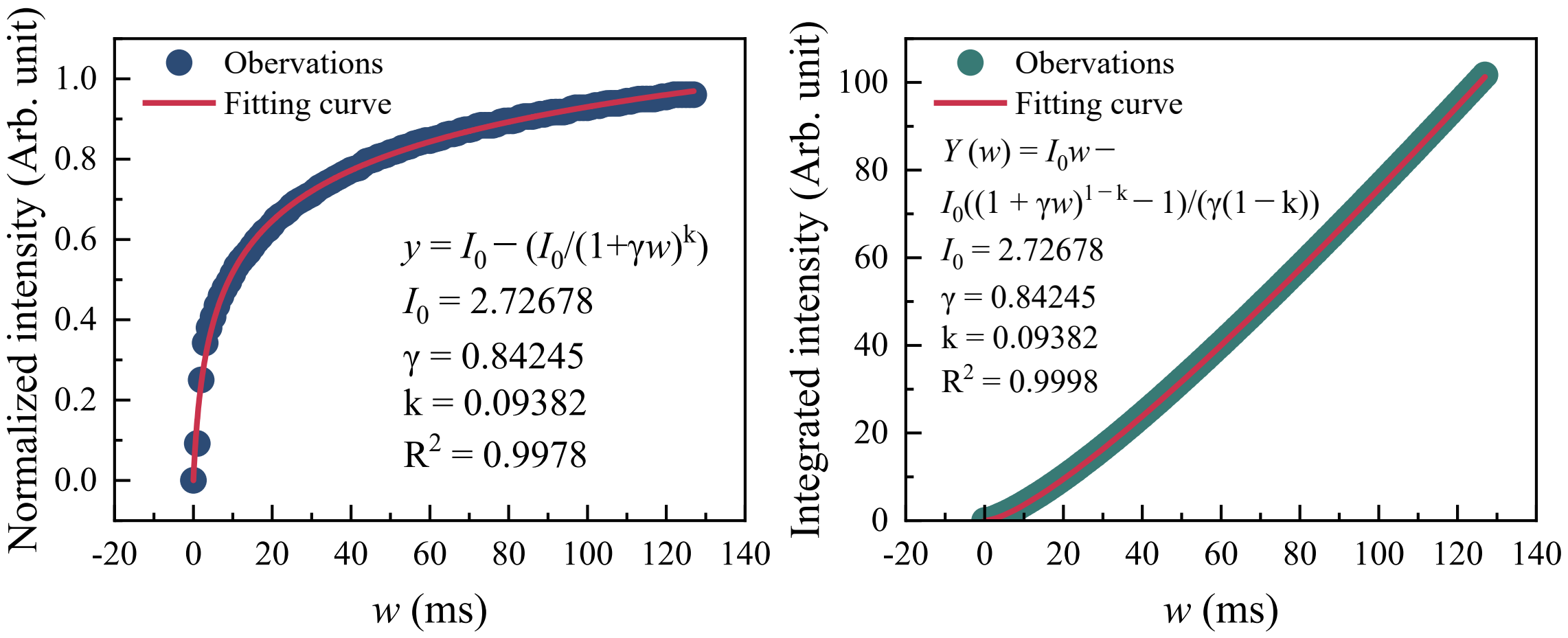

Figure S15. (a) Time dependent luminescence intensity curve of MGO@PDMS at $w = 0 – 125$ ms. (b) Time dependent integrated luminescence intensity curve of MGO@PDMS at $w = 0 – 125$ ms.

References

1 Hirschle, C., Schreuer, J. & Galazka, Z. Interplay of cation ordering and thermoelastic properties of spinel structure MgGa2O4. *Journal of Applied Physics* **124** (2018).

2 Jiang, F. *et al.* Temperature-induced phase transitions for stuffed tridymites SrGa2O4 and CaGa2O4. *Journal of Solid State Chemistry* **254**, 195-199 (2017).

3 Kahlenberg, V., Fischer, R. X. & Parise, J. B. The Stuffed Framework Structure of BaGa2O4. *Journal of Solid State Chemistry* **154**, 612-618 (2000).

4 Rietveld, H. Line profiles of neutron powder-diffraction peaks for structure refinement. *Acta Crystallographica* **22**, 151-152 (1967).

5 Lutterotti, L., Matthies, S., Wenk, H. R., Schultz, A. S. & Richardson, J. W. Combined texture and structure analysis of deformed limestone from time-of-flight neutron diffraction spectra. *Journal of Applied Physics* **81**, 594-600 (1997).

6 von Szczepanski, J. *et al.* High-Permittivity Polysiloxanes for Bright, Stretchable Electroluminescent Devices. *Advanced Optical Materials* **12**, 2400132 (2024).

7 Rendeiro, R., Jargus, J., Nedoma, J., Martinek, R. & Marques, C. The possibilities of using a mixture of PDMS and phosphor in a wide range of industry applications. *Opto-Electronic Advances* **7**, 240133-240131-240133-240121 (2024).

8 Sharma, T., Arora, G., Ng, C. Y. & Jun, H. K. A short review on long persistent luminescence materials and their application prospects in emerging photovoltaic devices. *Heliyon* **10**, e40341 (2024).

9 Huntley, D. J. An explanation of the power-law decay of luminescence. *Journal of Physics: Condensed Matter* **18**, 1359 (2006).

10 Sahai, M. K., Bakshi, A. K. & Datta, D. Revisit to power law decay of luminescence. *Journal of Luminescence* **195**, 240-246 (2018).

11 Van den Eeckhout, K., Bos, A. J. J., Poelman, D. & Smet, P. F. Revealing trap depth distributions in persistent phosphors. *Physical Review B* **87**, 045126 (2013).

12 Kai, H.-Y., Wong, K.-L. & Tanner, P. A. Tunneling in Persistent Luminescence. *Advanced Optical Materials* **13**, 2500793 (2025).

13 Van den Eeckhout, K., Smet, P. F. & Poelman, D. Persistent Luminescence in Eu2+-Doped Compounds: A Review. *Materials* **3**, 2536-2566 (2010).

14 Abeywickrama, T. M., Guo, A. & Mao, Y. Trap-Engineering the Persistent Luminescence of Ca3Ga4O9: Tb3+ via Al3+ Substitution for Optical Data Storage. *Advanced Optical Materials* **13**, 2402353 (2025).

15 Wang, S. *et al.* X-ray-activated super long persistent ultraviolet A luminescence from Ca3(PO4)2: Ce3+. *Journal of the American Ceramic Society* **107**, 4242-4251 (2024).

16 Peng, D. *et al.* All-Optical Synapses Based on a Mechanoluminescent Material. *Advanced Materials* **37**, 2503376 (2025).

17 Wi, S., Jeong, M., Lee, K. & Lee, Y. Optoelectronic Synapse Behaviors in Tb3+ and Al3+ Co-Doped CaSnO3 with Long-Persistent Luminescence. *Advanced Science* **11**, 2402848 (2024).

18 Wi, S. *et al.* Multi-Color Synaptic Luminescence in RE-Doped Ca2SnO4 (RE= Sm3+, Er3+, and La3+). *Advanced Functional Materials* **35**, 2414860 (2025).
